# Glycan-reactive antibodies isolated from human HIV-1 vaccine trial participants show broad pathogen cross-reactivity

**DOI:** 10.1128/jvi.01256-25

**Published:** 2025-11-10

**Authors:** Parker J. Jamieson, Xiaoying Shen, Alexandra A. Abu-Shmais, Perry T. Wasdin, Katarzyna Janowska, Robert J. Edwards, Garrett Scapellato, Maurice Bukenya, Lindsay E. Bass, Simone I. Richardson, Nelia P. Manamela, Shuying Liu, Maggie Barr, Lindsey Adams, Cristina Paola Velez-Castro, Caitlin McCarthy, Caroline A. Alexander, Rebecca A. Gillespie, Jessica Mimms, Naveenchandra Suryadevara, Ty A. Sornberger, Seth J. Zost, Robert Parks, Shelby Flaherty, Alexis K. Janke, Bethany N. Howard, Yukthi P. Suresh, Ruth M. Ruprecht, James E. Crowe, Robert H. Carnahan, Justin R. Bailey, Masaru Kanekiyo, Daniel Lingwood, Barton F. Haynes, Penny L. Moore, Rachel H. Bonami, Georgia D. Tomaras, Priyamvada Archarya, David C. Montefiori, Spyros A. Kalams, Shan Lu, Ivelin S. Georgiev

**Affiliations:** 1 Vanderbilt Center for Antibody Therapeutics, Vanderbilt University Medical Center, Nashville, Tennessee, USA; 2 Department of Pathology, Microbiology and Immunology, Vanderbilt University Medical Center, Nashville, Tennessee, USA; 3 Department of Surgery, Duke University School of Medicine 12277 , Durham, North Carolina, USA; 4 Program in Chemical and Physical Biology, Vanderbilt University Medical Center 12328, Nashville, Tennessee, USA; 5 Duke Human Vaccine Institute, Duke University School of Medicine 12277 , Durham, North Carolina, USA; 6 Department of Integrative Immunobiology, Duke University School of Medicine 12277 , Durham, North Carolina, USA; 7 Center for Human Systems Immunology, Duke University School of Medicine 12277 , Durham, North Carolina, USA; 8 South African Medical Research Council Antibody Immunity Research Unit, Faculty of Health Sciences, University of the Witwatersrand 37708 , Johannesburg, South Africa; 9 Centre for HIV and STIs, National Institute for Communicable Diseases of the National Health Laboratory Services, Johannesburg, South Africa; 10 Worcester HIV Vaccine, Worcester, Massachusetts, USA; 11 Ragon Institute of Mass General, MIT, and Harvard 200750 , Cambridge, Massachusetts, USA; 12 Vaccine Research Center, National Institute of Allergy and Infectious Diseases, National Institutes of Health, Bethesda, Maryland, USA 35037, Bethesda, Maryland, USA; 13 Division of Infectious Diseases, Department of Medicine, Johns Hopkins University School of Medicine 1500, Baltimore, Maryland, USA; 14 Texas Biomedical Research Institute and Southwest National Primate Research Center 7075, San Antonio, Texas, USA; 15 Department of Medicine, Duke University School of Medicine 12277 , Durham, North Carolina, USA; 16 Department of Integrative Immunology, Duke University School of Medicine 12277 , Durham, North Carolina, USA; 17 Vanderbilt Institute for Infection, Immunology and Inflammation, Vanderbilt University Medical Center 12328, Nashville, Tennessee, USA; 18 Department of Medicine, Division of Rheumatology and Immunology, Vanderbilt University Medical Center 12328, Nashville, Tennessee, USA; 19 Vanderbilt Center for Immunobiology, Vanderbilt University Medical Center 12328, Nashville, Tennessee, USA; 20 Department of Surgery, Duke University School of Medicine 12277 , Durham, North Carolina, USA; 21 Duke Human Vaccine Institute, Duke University School of Medicine 12277 , Durham, North Carolina, USA; 22 Department of Biochemistry, Duke University School of Medicine 12277 , Durham, North Carolina, USA; 23 Infectious Diseases Unit, Department of Internal Medicine, Vanderbilt University Medical Center 12328, Nashville, Tennessee, USA; 24 Department of Microbiology and Immunology, Vanderbilt University Medical Center 12328, Nashville, Tennessee, USA; 25 Department of Computer Science, Vanderbilt University 5718, Nashville, Tennessee, USA; 26 Center for Structural Biology, Vanderbilt University 5718, Nashville, Tennessee, USA; 27 Center for Computational Microbiology and Immunology, Vanderbilt University Medical Center 12328, Nashville, Tennessee, USA; Icahn School of Medicine at Mount Sinai, New York, New York, USA

**Keywords:** human immunodeficiency virus, vaccines, monoclonal antibodies, LIBRA-seq, glycan-reactive, human clinical trials, bNAb, cross-reactive, Fab-dimerized, hepatitis C virus

## Abstract

HIV-1 continues to pose a significant global health challenge, requiring ongoing research into effective prevention and treatment strategies. Understanding the B-cell repertoire that can be engaged upon vaccination in humans is crucial for the development of future preventive vaccines. In this study, peripheral blood mononuclear cells from HIV-negative participants in the multivalent HVTN124 human HIV-1 vaccine clinical trial were interrogated for HIV-reactive B cells using LIBRA-seq, a high-throughput B-cell mapping technology. We report the discovery of glycan-reactive antibodies, with one of these being capable of neutralizing diverse heterologous HIV-1 virus strains. Furthermore, isolated antibodies showed broad cross-reactivity against antigens from a variety of other pathogens. The emerging class of glycan-reactive virus-neutralizing antibodies with exceptional breadth of pathogen cross-reactivity may present an effective target for vaccination at the population level.

**IMPORTANCE:**

Understanding how the human immune system recognizes and combats viruses is crucial for developing better vaccines and treatments. Here, through characterization of the B-cell receptor repertoires of participants in HVTN124, a multivalent HIV-1 vaccine human clinical trial, we discovered antibodies that recognize sugar molecules (glycans) on antigens from a range of unrelated viral families. In addition to their binding breadth, these antibodies can also neutralize multiple diverse strains of HIV-1. Our findings reveal an emerging and underappreciated mechanism for antibodies to counteract virus infection, potentially opening doors for developing vaccines that preferentially elicit glycan-reactive antibody species to broadly protect against different viruses.

This study is registered with ClinicalTrials.gov as NCT03409276.

## INTRODUCTION

Human immunodeficiency virus type 1 (HIV-1) is a positive-sense single-stranded (ssRNA) retrovirus that affects 38.4 million people globally and is the causative agent for acquired immunodeficiency syndrome (AIDS). Once transmitted via bodily fluids, HIV-1 will target CD4+ cells and integrate into the host cell genome. Integration allows for the constant production of new HIV-1 virions; however, this is met by a strong adaptive immune response to reduce the total viral load. In this tug-of-war between HIV-1 and the immune system, HIV-1 can create viral escape mutants that increase viral diversity and the potential to evade the host’s adaptive immune response (1–7). A common source of escape exists in the surface viral envelope (Env) gene, which encodes the gp160 envelope glycoprotein trimer. The gp160 trimer allows HIV-1 to target, bind, and enter CD4+ cells. The gp160 trimer is divided into two subunits: a gp120 receptor/co-receptor-binding protein and a gp41 transmembrane protein (1–7). The gp120 subunit comprises critical epitopes that influence HIV-1 infectivity and are primary antibody targets. Some of these epitopes include the CD4-binding site for initial binding to CD4+ cells, the V1/V2 loop for trimer stabilization, and the V3-glycan loop for co-receptor recognition. Because the gp160 trimer is exposed on the viral surface, it faces constant selective pressure by the adaptive immune system, resulting in frequent mutations that confer antibody evasion. In addition to sequence diversity, HIV-1 Env is comprised of a host-derived glycan shield that can mask critical epitopes from neutralizing antibodies. By mutating Env and other viral genetic components, along with displaying self-like carbohydrate structures, HIV-1 can diversify into distinct groups, clades, and present different gp160 epitopes, thus creating a major obstacle regarding HIV-1 vaccine development (1–7).

While effective vaccines, such as the mRNA COVID-19 vaccine, have mitigated many infectious diseases, there is still no effective preventive HIV-1 vaccine. Antiretroviral medications suppress viral replication effectively; however, individuals infected with HIV-1 continue to harbor the virus and have to take antiretroviral therapies (ART) throughout their lives and are subjected to long-term drug toxicity. The most promising HIV-1 vaccine trial to date was the RV144 trial in Thailand, which tested a canarypox vector prime-Env gp120 protein boost vaccine. However, the 31.2% efficacy of RV144 was not robust, and subsequent canarypox vector-Env gp120 protein vaccine trials in South Africa failed to show efficacy (8–16). The development of a safe and protective vaccine against HIV-1, though a formidable challenge, remains a worldwide health priority.

**Fig 1:**
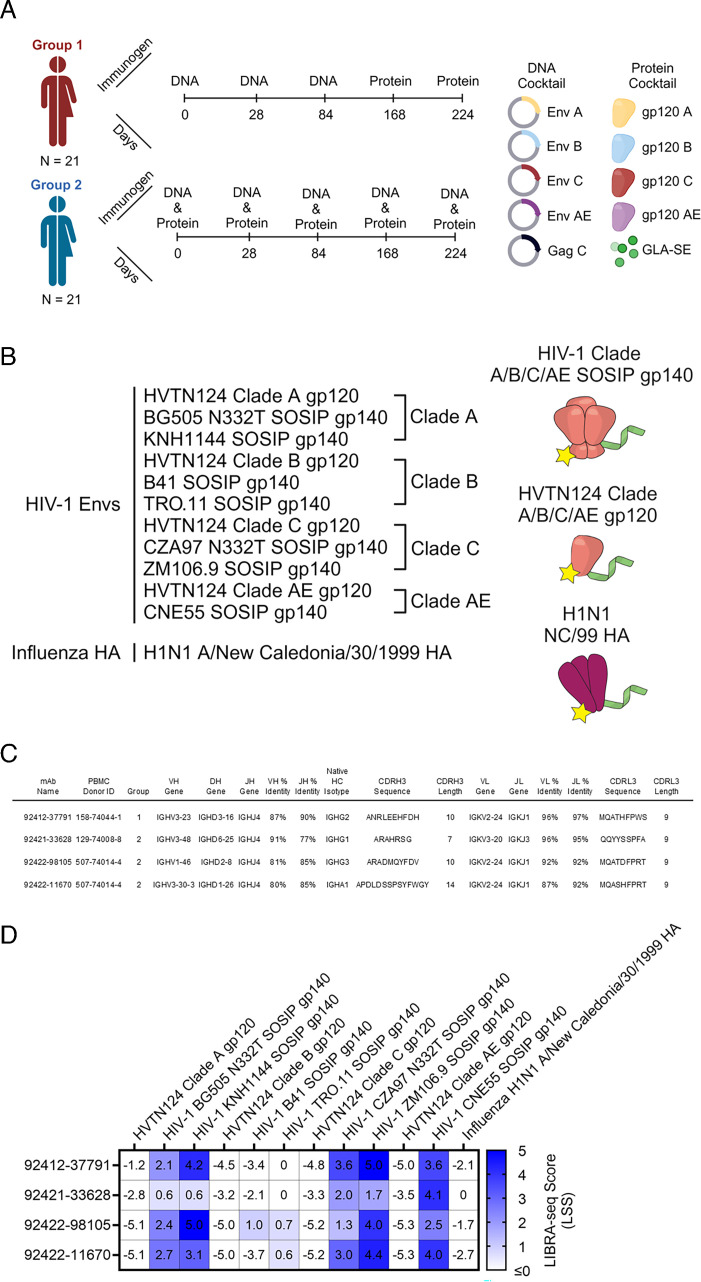
LIBRA-seq discovery of mAbs from HVTN124 donor PBMCs. (**A**) HVTN124 vaccine trial. Graphic of the HVTN 124 immunization groups, time points, and makeup. The figure was created using BioRender. (**B**) Antigen screening library used to interrogate the B-cell repertoire from HVTN124 donor PBMCs. The library consisted of four HVTN124 gp120 immunogens, seven SOSIP gp140 antigens, and one influenza HA antigen. Yellow stars represent phycoerythrin (PE), and the green ribbons represent the unique DNA barcodes. The figure was created using BioRender. (**C**) Sequence features of identified HIV-1 Env-reactive mAbs isolated from HVTN124 donor PBMCs. PBMC donor identity and vaccine group are also provided. All antibodies were expressed as IgG1 for further characterization. Additional sequence features can be found in Fig. S1. (**D**) Antigen-specific LIBRA-seq scores for the four HIV-1 Env-reactive mAbs (rows) are shown for the antigens used in the screening library (columns). Heatmap of LIBRA-seq scores ranges from white (low) to blue (high).

A challenge for HIV-1 vaccine design is eliciting effective HIV-1 antibodies that protect against a diverse range of viral variants. HVTN 124, developed by Worcester HIV Vaccine, was a phase 1A human vaccine trial that was completed in October 2020 and utilized a second-generation polyvalent DNA–protein HIV vaccine consisting of DNA inserts expressing four HIV-1 gp120 antigens and an HIV-1 gag gene, and the four matching adjuvanted recombinant HIV-1 gp120 proteins in both prime–boost and coadministration regimens aiming to elicit high magnitude, cross-reactive antibodies against multiple HIV-1 clades (17). Immunogens used included HIV-1 Env gp120 DNA and proteins from clades A (92UG037.8), B (JR-FL), C (93MW965.26), and AE (ConAE [Ref: 92KDSu5-19150]), along with DNA of the conserved HIV-1 Gag polyprotein and a glucopyranosyl lipid adjuvant in a stable emulsion (GLA-SE) to stimulate a Th1-dominant response (18–21). The HVTN 124 clinical trial included a total of 60 HIV-negative men and women across different age ranges and ethnic backgrounds. Participants were randomly divided into three groups: Group 0 for establishing safety, Group 1 for staggered immunizations, and Group 2 for concurrent immunizations. A schematic of the HVTN124 immunization strategies, groups, and composition is depicted in Fig. 1A. Preclinical experiments in rhesus macaques, mice, and rabbits identified that staggered immunizations (Group 1 approach) elicited antibodies with the highest avidity, high titers, and broad reactivity (22–27). In one of these preclinical animal studies, the HVTN124 vaccine composition and staggered immunizations in rabbits elicited high IgG titers against the V1/V2 epitope, which is responsible for trimer stabilization, neutralization resistance, and modulates viral entry (27). In the HVTN 124 trial, Groups 1 and 2 both elicited polyclonal cross-reactive HIV-1 antibodies in serum; however, the monoclonal antibody response had yet to be characterized for either group. Monoclonal antibody characterization is essential for HIV-1 vaccine design as it informs features of antibodies that may help define their effectiveness. These include the ability of a vaccine to elicit antibodies that recognize diverse epitopes across inter- and intra-clade HIV-1 strains, or cross-react and neutralize the virus.

Here, we report the isolation and characterization of a set of glycan-reactive, including Fab-dimerized glycan-reactive (FDG), antibodies from HVTN124 trial participants that broadly neutralize HIV-1 and hepatitis C virus (HCV). FDGs, such as the potent 2G12 broadly neutralizing antibody (bNAb) that utilizes a unique V_H_ domain swapping configuration resulting in Fab dimerization that presents as I-shaped, along with others such as DH851 that Fab dimerize without V_H_ domain swapping, have been previously reported in the literature as targeting conserved glycan patches (28). A subset of these antibodies was found to be Fab-dimerized by negative-stain electron microscopy (NSEM) analysis. Moreover, some of these antibodies share public sequence signatures, or recurring complementarity-determining region (CDR) sequence motifs, with other known glycan-reactive antibodies, suggesting that such a public class of antiviral antibody recognition can be used as a target for broadly reactive vaccine candidates against HIV-1. We demonstrate that of the four antibodies isolated from HVTN124 peripheral blood mononuclear cells (PBMCs) that bind to N-linked glycans, three of the four neutralized HIV-1, with one showing weak but broad neutralization across HIV-1 viruses from tiers 1 and 2. Furthermore, we show that these antibodies cross-react with antigens from multiple diverse pathogens, including neutralization against HCV, but are mostly negative on autoreactivity assays. Overall, we propose that the elicitation of glycan-reactive antibodies can serve as an effective strategy for turning the elusive HIV-1 glycan shield into a Trojan horse, and therefore warrants further pursuit and optimization in future HIV-1 vaccine trials.

## RESULTS

### Isolation of HIV-reactive antibodies from HVTN124 participants

**Fig 2:**
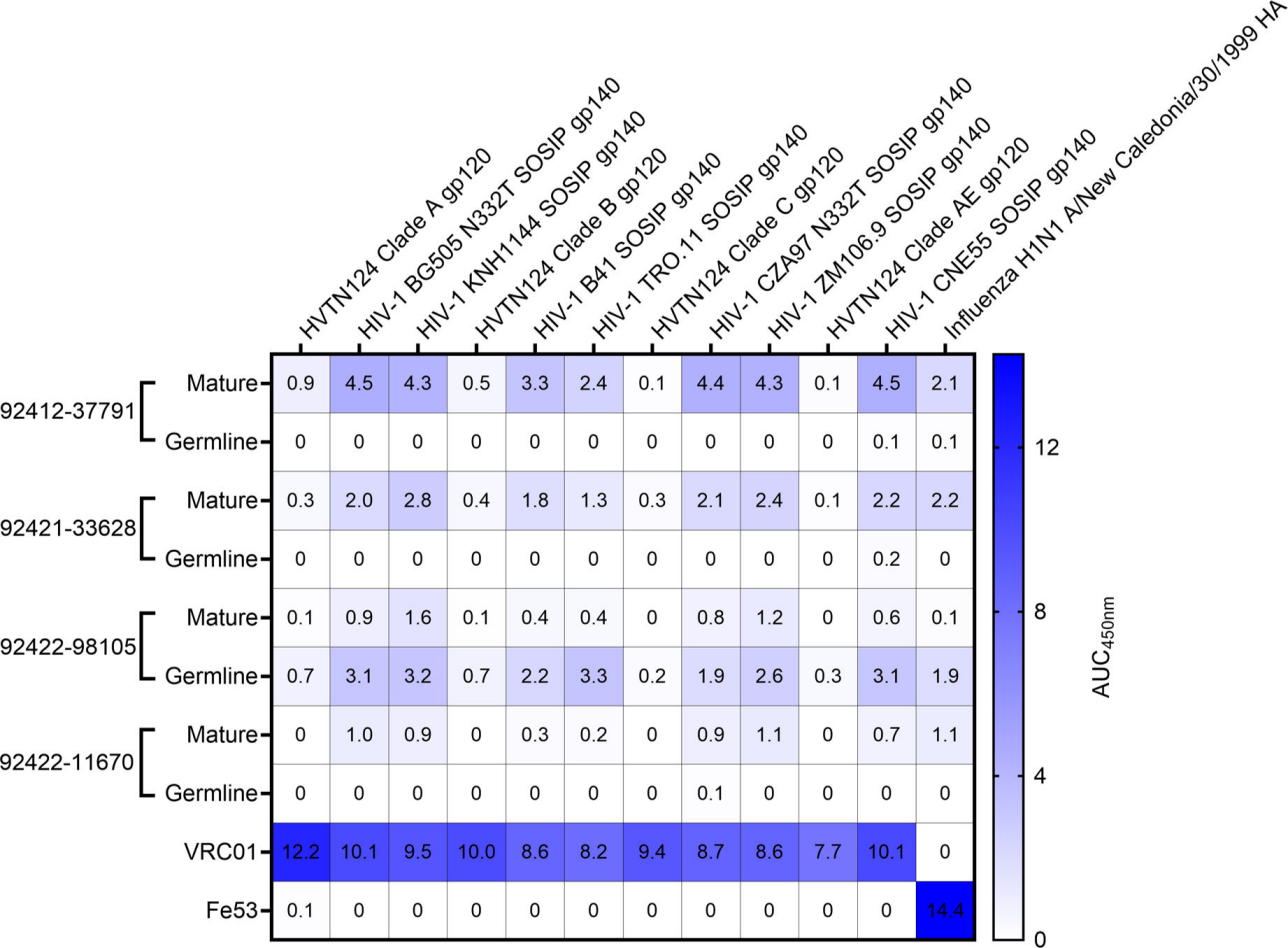
Characterization of HVTN124 mAbs by ELISA. ELISA validation of HVTN124 mAbs displayed as area under the curve (AUC) for the 12-antigen screening panel (columns) versus antibodies (rows), including the four HVTN124 mAbs, VRC01 (HIV-1 positive control), and Fe53 (influenza positive control). Values for all conditions were gathered in duplicate from a set of three repeats.

To identify HIV-reactive B cells from HVTN124 participants, we performed LIBRA-seq, a high-throughput single-cell sequencing technology (29). The LIBRA-seq antigen screening panel consisted of the four autologous HVTN124 gp120 vaccine immunogens (HVTN124 gp120 A, HVTN124 gp120 B, HVTN124 gp120 C, and HVTN124 gp120 AE) and seven heterologous SOSIP gp140 antigens (BG505, KNH1144, B41, TRO.11, CZA97, ZM106.9, and CNE55). Influenza H1N1 A/New Caledonia/30/1999 hemagglutinin (HA) was also included in the antigen screening panel as a negative control. HIV-1 SOSIP gp140 antigens were stabilized by an intermolecular disulfide bond between gp120 and gp41, displayed a truncated gp41 transmembrane region, and contained a variable flexible linker between gp120 and gp41 (30–35). This 12-antigen panel, shown in Fig. 1B, was applied separately to individual PBMC samples from three donors, resulting in the isolation of B cells with varying degrees of predicted cross-reactivity between multiple HIV-1 antigens. Notably, several B-cell receptors (BCRs), isolated from multiple different HVTN124 participants, showed cross-reactivity between multiple HIV-1 antigens, with stronger signals for the SOSIP variants compared to the gp120 vaccine antigens, and binding was also observed for the influenza HA antigen (Fig. 1C and 2; Fig. S1 and S2). Three of the four HVTN124 mAbs exhibited increased binding strength from their inferred germline to mature forms, consistent with the effects of somatic hypermutation (SHM) during affinity maturation (36, 37). However, 92422–98105 deviated from this trend by demonstrating stronger binding in its germline form than its mature counterpart, suggesting that SHM may have led to reduced binding cross-reactivity for this antibody. The preferential binding to SOSIP vs. gp120 vaccine antigens was in accordance with LIBRA-seq scores for the respective antigens (Fig. 1D). Given the intriguing cross-reactivity with the influenza H1N1 A/New Caledonia/30/199 hemagglutinin (HA) antigen, we characterized these antibodies further.

### HVTN124 antibodies target N-linked glycans on the surface of HIV-1 Env

**Fig 3:**
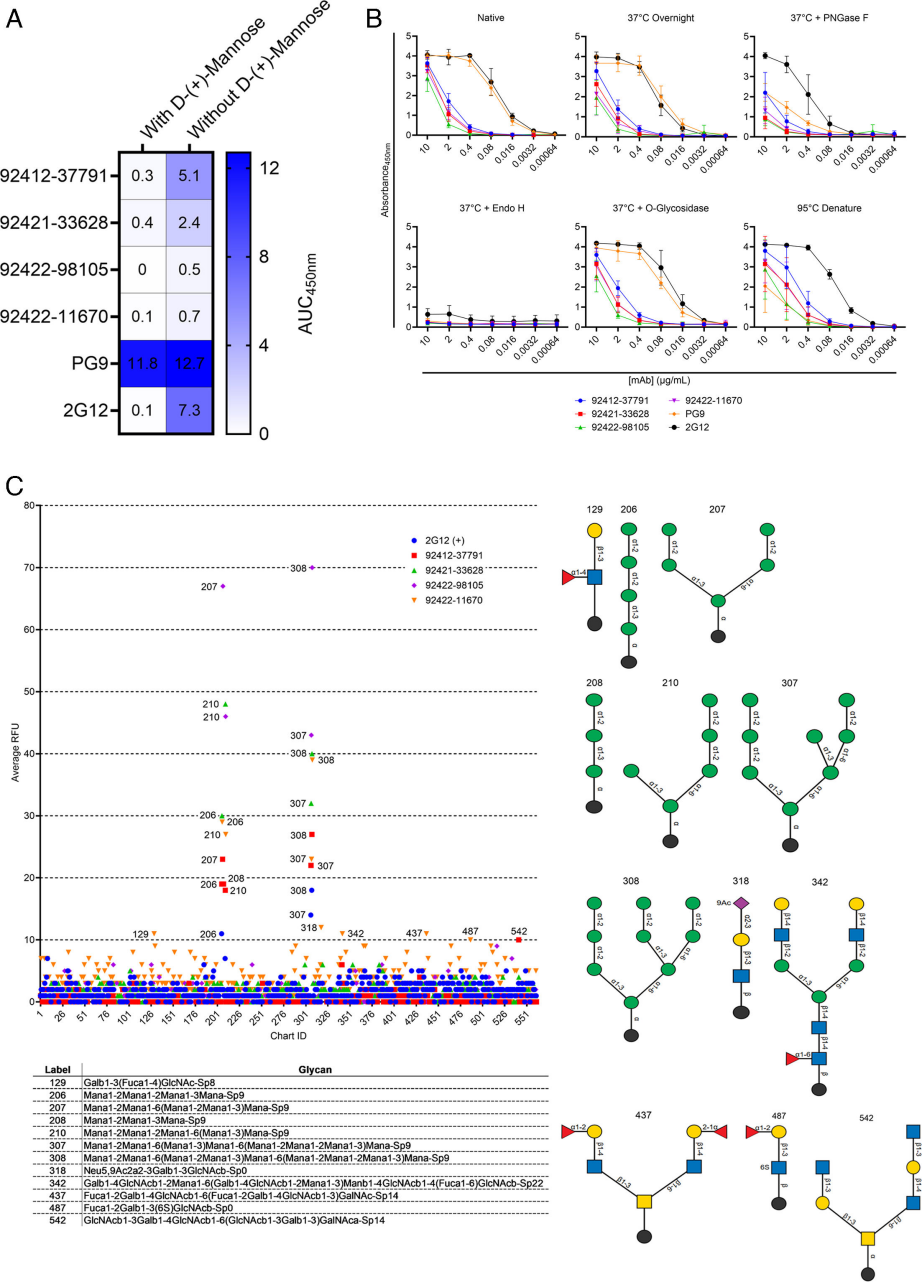
HVTN 124 mAbs achieve broad reactivity via N-linked glycan recognition. (**A**) Antibody binding with and without 1M D-(+)-Mannose competition is displayed as a heatmap. The four HVTN124 mAbs, along with the V3-glycan-reactive 2G12 and V1/V2-reactive PG9 control mAbs (rows), were incubated with and without 1M D-(+)-Mannose against CNE55 (columns). Results are displayed from a set of three repeats in duplicate. (**B**) Enzymatic deglycosylation of HIV-1 CNE55 SOSIP gp140 tested by ELISA. The glycan-dependency of the HVTN124 mAbs was further evaluated by treating HIV-1 CNE55 SOSIP gp140 with different deglycosylation and/or denaturing conditions, then testing by ELISA. X-axis lists the antibody concentration in µg/mL, while absorbance at 450 nm is listed along the Y-axis. Values for all six conditions are displayed from a set of three repeats in duplicate. (**C**) Glycan microarray. Biotinylated HVTN124 mAbs and the 2G12-positive control mAb were tested against a panel of 562 glycan structures at 50 μg/mL. Antibody binding was first detected using anti-human IgG-biotin at 5 μg/ml, then followed by a second detection step using streptavidin-Cy5 at 0.5 μg/mL. Data are shown as the average relative fluorescence units (RFUs). Values at and above 10 RFU are considered a positive signal. The chemical nomenclature for glycan structures with positive results is listed below, with their structures displayed on the right. Diagrams of glycan structures were generated using GlycoGlyph (49). Glycan microarray protocol was followed using the microwave-assisted wet-erase process by Mehta et al. (50). The assay was performed in replicates of six.

After observing strong reactivity to influenza H1N1 A/New Caledonia/30/1999 HA by ELISA, we sought to identify what common features exist between HIV-1 Env and H1N1 Influenza HA. Since both antigens are glycoproteins, we elected to determine whether these antibodies may target N-linked glycans as a common epitope. N-linked glycosylation sites on HIV-1 Env comprise roughly 50% of its molecular mass, with 70%–90% of glycans on Env being forms of high mannose (38–40). These host-derived N-linked glycans on Env have been established to form a glycan shield that helps HIV-1 evade NAbs by masking potentially neutralizing epitopes, along with ensuring proper Env folding and infectivity (41). One of the most mannose-dense domains of HIV-1 Env lies at the third variable loop, or V3-glycan (42). The V3-glycan is crucial for HIV-1 Env tropism and binding to the CCR5 or CXCR4 coreceptors, making conserved domains of the V3-glycan one of the primary targets for human glycan-reactive HIV-1 neutralizing antibodies (41, 43–46). Seasonal influenza HA glycoproteins, which include H1N1, H3N2, and Victoria lineages, have been found to contain roughly 28%–50% of high-mannose glycans; however, the proportion of high-mannose glycans on influenza HA can vary between strains (47). Since HIV-1 Env and influenza HA both contain high-mannose glycans, we sought to test for antibody competition against D-(+)-Mannose, serving as a common glycan target between both viral antigens. As seen in Fig. 3A and Fig. S3, when incubated with 1M D-(+)-Mannose, all four of the HVTN124 mAbs and 2G12 showed a noticeable decrease in binding to HIV-1 CNE55 SOSIP gp140 by ELISA, whereas the V1/V2-reactive PG9 antibody, which recognizes gp120-associated high-mannose glycans but will not bind to protein-free glycans, showed no significant decrease in reactivity toward HIV-1 CNE55 SOSIP gp140 (48).

To further validate that these four HVTN124 mAbs bind to N-linked glycans, we performed a series of enzymatic deglycosylation reactions against HIV-1 CNE55 SOSIP gp140. Specific conditions of HIV-1 CNE55 SOSIP gp140 included the following: native, 37°C overnight, 37°C overnight with PNGase F, 37°C overnight with Endo H, 37°C with O-glycosidase, and denatured at 95°C for 10 minutes. While the enzymes PNGase F and Endo H both cleave N-linked glycans from glycoproteins, PNGase F removes all high-mannose, complex, and hybrid N-linked glycans, whereas Endo H only removes high-mannose and some hybrid N-linked glycans. It has been previously reported that when the CHO-cell-expressed HIV-1 gp120 protein is treated with Endo H, the binding affinity of 2G12 is significantly reduced due to the removal of high-mannose carbohydrates (51). O-glycosidase only removes desialylated core 1 and core 3 O-linked disaccharides that are attached to serine or threonine residues. While not as extensive as N-linked glycans, O-linked glycans have been previously reported on a subset of HIV-1 isolates with unusually long V1 domains and can aid in shielding HIV-1 from V3-glycan bNAbs (52).

As shown in Fig. 3B, the PNGase F and Endo H N-linked deglycosylation conditions displayed a noticeable reduction in antibody binding activity to HIV-1 CNE55 SOSIP gp140. Since HIV-1 can display O-linked glycans on the Env surface glycoprotein, we also opted to include the O-glycosidase O-linked deglycosylation condition; however, as shown in Fig. 3B, there was no noticeable change in binding levels toward the HIV-1 CNE55 SOSIP gp140 with O-linked glycans removed. The 95°C denaturing condition was included to address whether proper protein structure is necessary for antibody binding, which was shown not to be the case for each of the HVTN124 mAbs.

We next performed glycan microarray analysis to identify the specific N-linked glycan structures recognized by the HVTN124 mAbs. All HVTN 124 mAbs and the 2G12 positive control displayed affinities toward linear and branched high-mannose glycan structures (Fig. 3C). Unlike 2G12 and the other HVTN 124 mAbs, 92412-37791 and 92422-11670 also displayed affinities toward complex and hybrid glycan structures; however, these were only slightly at or above the positive threshold of 10 RFU. These data indicate that the four HVTN124 mAbs are reactive to N-linked glycans on HIV-1 Env, specifically D-(+)-Mannose.

### HVTN 124 antibodies show different levels of reactivity with intracellular and extracellular autoantigens

**Fig 4:**
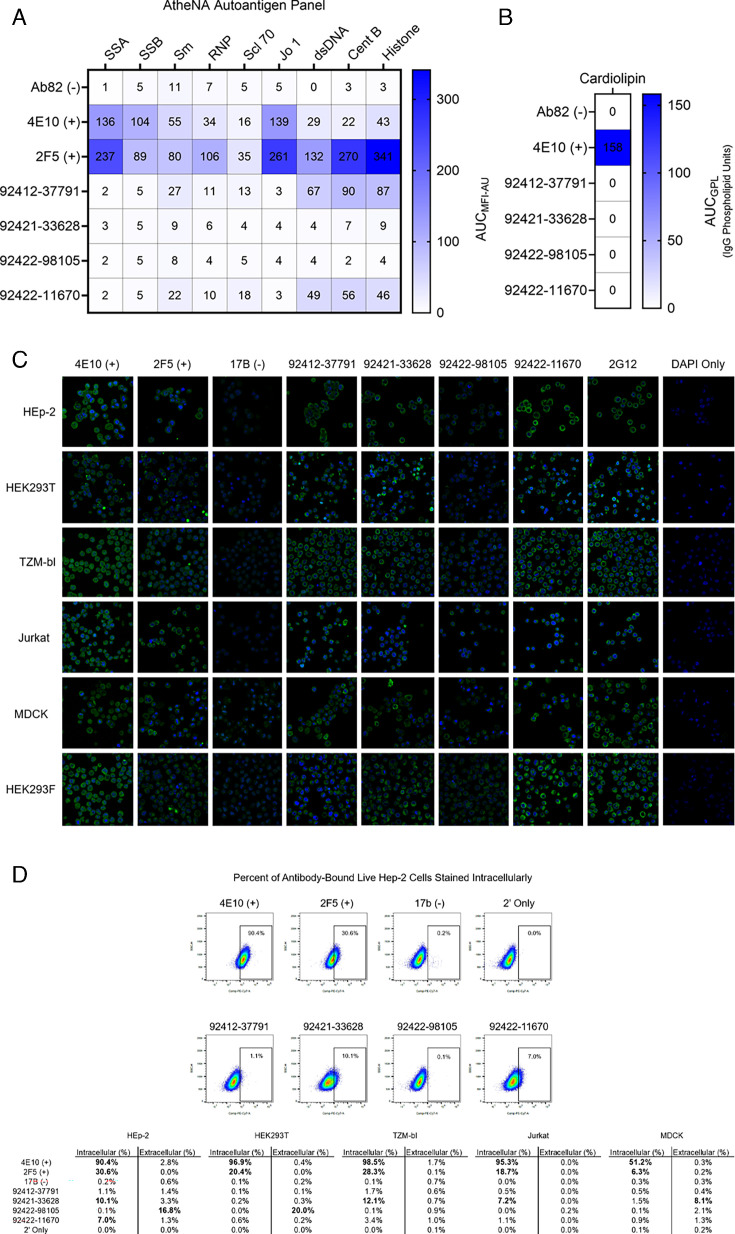
Autoreactivity analysis of HVTN124 mAbs. (**A**) Autoantigen reactivity against the AtheNA panel. Autoantigens are listed as columns, while antibodies are listed as rows. Positive control antibodies included 4E10 and 2F5, while Ab82 was used as a negative control antibody. In reference to Fig. S3, values exceeding 120 MFI at 25 µg/mL for the AtheNA assay are considered positive. Values for all conditions are displayed from a set of two repeats. (**B**) Autoantigen reactivity against cardiolipin. Antibodies are listed as rows. 4E10 was used as a positive control antibody, while Ab82 was used as a negative control antibody. In reference to Fig. S4, values at or greater than 20 GPL (IgG Phospholipid Units) at 50 µg/mL are considered positive. Values for all conditions are displayed from a set of two repeats. (**C**) Cell monolayer immunofluorescence. HVTN124 mAbs were applied to whole, permeabilized, uninfected HEp-2, HEK293T, TZM-bl, Jurkat, MDCK, and HEK293F cells at 25 µg/mL mAb. Antibodies were detected using 1:500 of goat anti-human IgG-AF488 (green), and counterstained with 1:5 of DAPI (blue). 4E10 and 2F5 were used as positive control mAbs, while 17b was used as a negative control mAb. A DAPI-only condition was also gathered to detect any autofluorescence. Images are at 40× magnification and were gathered in triplicate. (**D**) Flow cytometry determination of intracellular and extracellular self-antigen recognition by HVTN124 mAbs. Top: Representative flow cytometry plots for intracellular staining of HEp-2 cells with 25 µg/mL positive control mAbs 4E10 and 2F5, negative control mAb 17b, or experimental mAbs 92412-37791, 92421-33628, 92422-98105, and 92422-11670. Anti-human IgG Fc-PE/Cy7 was used to detect experimental and control recognition of cellular autoantigens. Bottom: Summary tables of percent antibody-bound HEp-2, HEK293T, TZM-bl, Jurkat, and MDCK live cells stained with mAbs intracellularly and extracellularly and gated as in the plots above. Positive cutoff for autoreactivity set at 5% (bold).

Next, we evaluated whether the four glycan-reactive antibodies display autoreactivity toward a panel of self-antigens. Three of the four antibodies showed no signal for any of the antigens in a standard AtheNA autoantigen panel of nuclear proteins, with only antibody 92412-37791 displaying any degree of autoreactivity for Centromere B and Histone (Fig. 4A and Fig. S4). When tested against cardiolipin, none of the four glycan-reactive antibodies displayed reactivity (Fig. 4B and Fig. S5). Next, we screened for reactivity against HEp-2, HEK293T, TZM-bl, Jurkat, MDCK, and HEK293F cells using cell monolayer immunofluorescence and analytical flow cytometry. Cell monolayers for the four HVTN124 mAbs displayed varying strength for each cell line (Fig. 4C). To determine the potential for recognition of intracellular vs. extracellular antigens expressed by these several cell lines (with the exception of HEK293F cells), we tested each of the four HVTN124 mAbs in flow cytometry assays (with gating defined in Fig. S6). Resulting data indicated that the HVTN124 mAbs display different levels of autoreactivity: 92412-37791 was not autoreactive against any of these cell lines; 92421-33628 displayed intracellular reactivity against HEp-2, TZM-bl, and Jurkat cells, in addition to extracellular reactivity against MDCK cells; 92422-98105 only showed extracellular reactivity against HEp-2 and HEK293T cells; and 92422-11670 only displayed intracellular reactivity against HEp-2 cells (Fig. 4D). However, the percent positivity of the HVTN124 mAbs was noticeably lower than that of the positive control mAbs, especially 4E10. Together, these findings indicate that while the HVTN124 glycan-reactive mAbs are autoreactive, their overall reactivity toward self-antigen is limited, suggesting that this antibody class is favorable and safe for further elicitation in future HIV-1 vaccine clinical trials.

### A subset of HVTN124 antibodies displays Fab-dimerization

**Fig 5:**
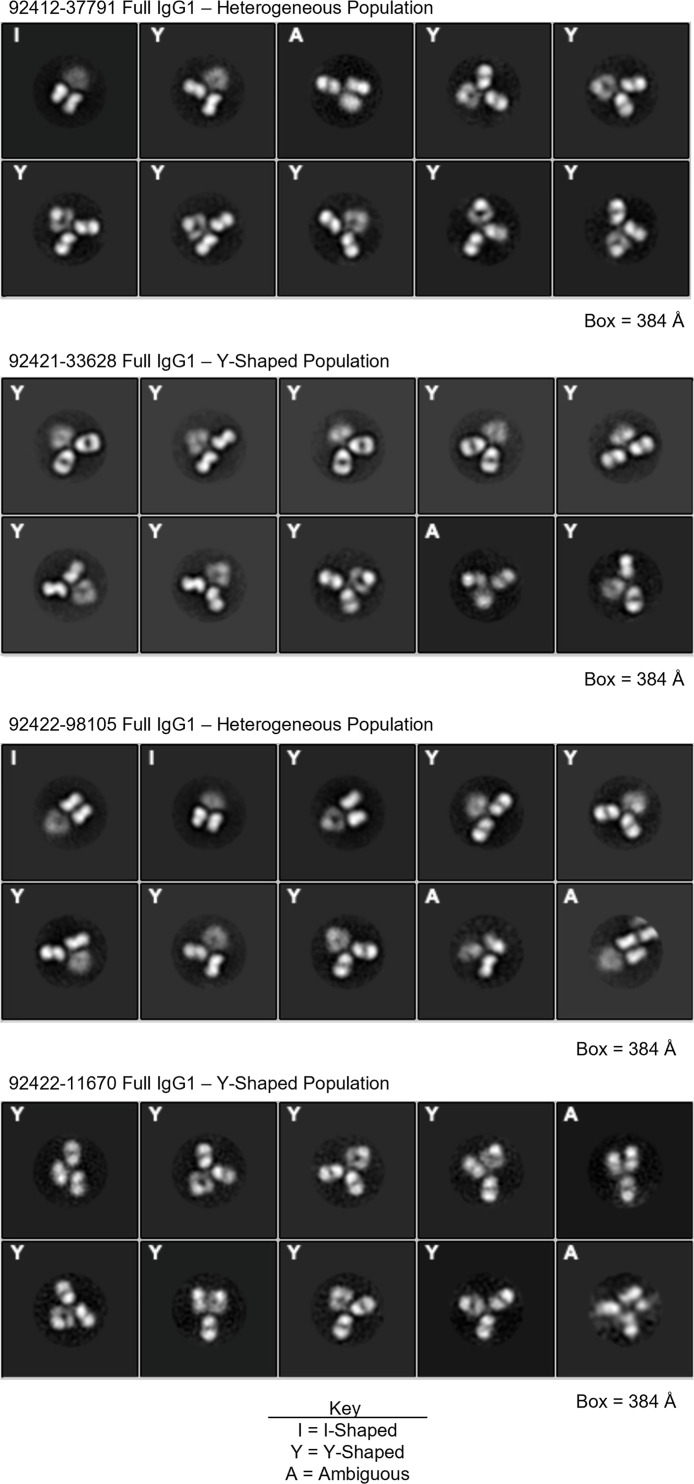
NSEM. NSEM 2D class averages of full IgG1 of 92412-37791 (top), 92421-33628 (2nd from top), 92422-98105 (2nd from bottom), and 92422-11670 (bottom). Imaging revealed that 92412-37791 and 92422-98105 are a heterogeneous population of I- and Y-shapes, while 92421-33628 and 92422-11670 are only Y-shaped. Images are ordered from most (top left corner) to least (bottom right corner) frequently observed by NSEM.

It has been previously reported that glycan-reactive antibody species can be presented in a Fab-dimerized I-shaped conformation (28). In HIV-naive humans, FDGs are encoded by diverse antibody variable genes, with IGHV3-23 (24.3%), IGHV1-46 (18.9%), IGKV1-5 (10.8%), and IGKV2-24 (45.9%) being among the most frequent (28). Interestingly, three of the four HVTN124 mAbs also utilized the IGKV2-24 light chain germline gene. To assess whether the HVTN124 mAbs are FDGs, we imaged the four HVTN124 mAbs as full IgG1 by NSEM. We observed a heterogeneous mix of I- and Y-shaped full IgGs for 92412-37791 and 92422-98105, while no I-shaped forms were observed for 92421-33628 and 92422-11670 (Fig. 5). The Fab dimerized configuration of the two I-shaped mAbs was most reminiscent of the previously published DH1005 antibody (28).

### Broad reactivity against multiple diverse viral antigens

**Fig 6:**
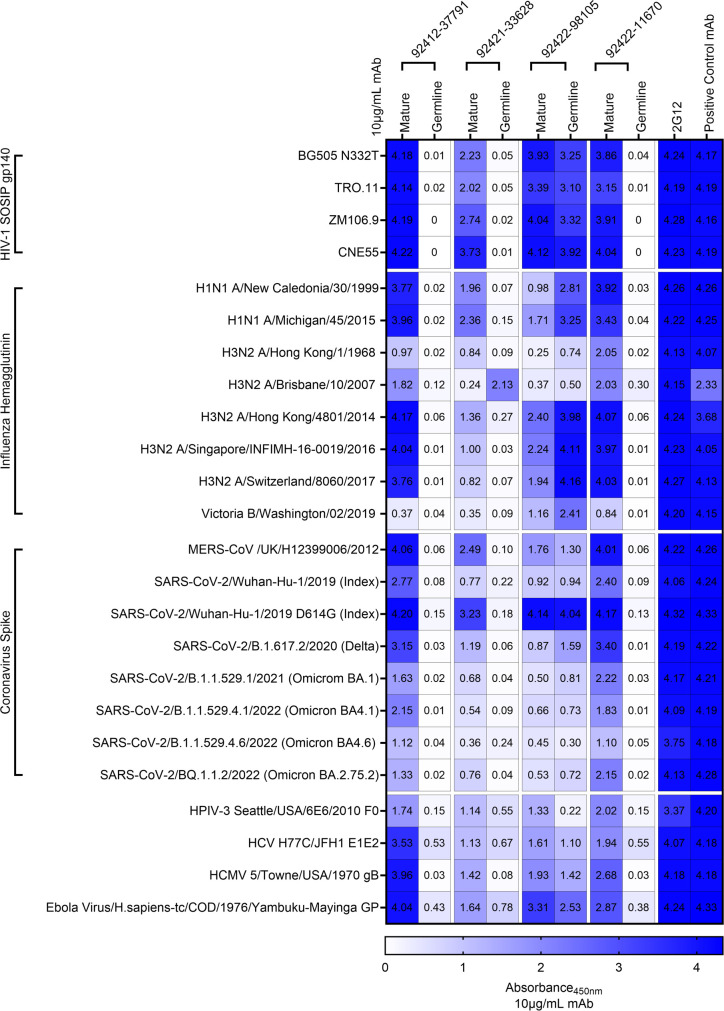
Expanded breadth analysis. HVTN 124 mature and germline IgG1 mAbs vs. 22-antigen breadth panel. Four HVTN 124 mAbs, 2G12, and the control mAbs are listed as columns, with the antigens used being listed as rows. Heatmap of absorbance values at 450 nm (A450 nm) using 10 µg/mL mAb is listed on the right from 0 in white to 4 in dark blue, with larger values in blue correlating to stronger antibody-antigen interactions. Control mAbs for each viral family include: VRC01 = HIV-1, CR9114 = influenza, 54043-5 = MERS CoV and SARS-CoV-2, 3 × 1 = HPIV3, AP33 = HCV, IG2 = HCMV, mAb114 = Ebola Virus. Values for all conditions were gathered in duplicate from a set of three repeats.

Following the observation that the HVTN124 glycan-reactive antibodies broadly react to HIV-1 Env and influenza H1N1 A/New Caledonia/30/1999 HA, we evaluated whether these antibodies could recognize a broader antigen panel from a variety of other viruses. The 22 antigens used in this breadth panel were from HIV-1 (4 SOSIP gp140), influenza (2 group 1 HA, 5 group 2 HA, and 1 type B HA), coronavirus (6 SARS-CoV-2 spike variants and MERS-CoV spike), human parainfluenza virus type 3 (HPIV3) F0, HCV E1E2, and human cytomegalovirus (HCMV) gB (Fig. 6). Each of the four HVTN124 mAbs showed broad reactivity against each of the viral families, albeit with variable binding strengths (Fig. 6). The antigens in this breadth panel contain a different number of N-linked glycan sites (on average: 26–30 (HIV-1 Env), 5–11 (influenza HA), 22 (SARS-CoV-2 spike), 5–6 (PIV3 F), 4–5 (HCV E1E2), and 18–19 (CMV gB), suggesting that these antibodies can target viral antigens with diverse glycan shield densities (53–58).

Next, we explored whether this level of broad reactivity was retained in germline-reverted versions of these antibodies, where both the heavy and the light chains for each antibody were reverted to their respective germline sequences apart from the CDR3 regions. Notably, the germline-reverted versions for three of the four antibodies showed virtually no or, in a small set, significantly reduced reactivity against the antigens tested (Fig. 6). The only exception was the germline-reverted version of antibody 92422-98105 that exhibited strong recognition toward several of the antigens in the panel, similar to or, in some cases, even better than the mature antibody (Fig. 6). Ramos B-cell lines were engineered to express either the mature or germline-reverted versions of 92421-33628 and 92422-98105 (Fig. S7). After initial flow cytometric analysis confirmed robust BCR surface expression, these Ramos B-cell lines were tested against HIV-1 KNH1144 SOSIP gp140 and HVTN124 Clade B gp120. A common trend observed for the mature Ramos cell lines was stronger binding to HIV-1 KNH1144 SOSIP gp140 than HVTN124 Clade B gp120, potentially due to gp120 Env being a monomeric form that could be missing glycan-dependent epitopes found on SOSIP gp140 Env. While still weak, the germline versions of 92421-33628 and 92422-98105 against HVTN124 Clade B gp120 were roughly 10-fold higher for the latter, suggesting that 92422-98105 was likely glycan-reactive as germline, while 92421-33628 became glycan-reactive via SHM. Together, these results indicate that the breadth of antigen recognition for three of the four glycan-reactive antibodies is associated with acquired SHM.

### Publicness of HVTN124 glycan-reactive antibody sequences

**Fig 7:**
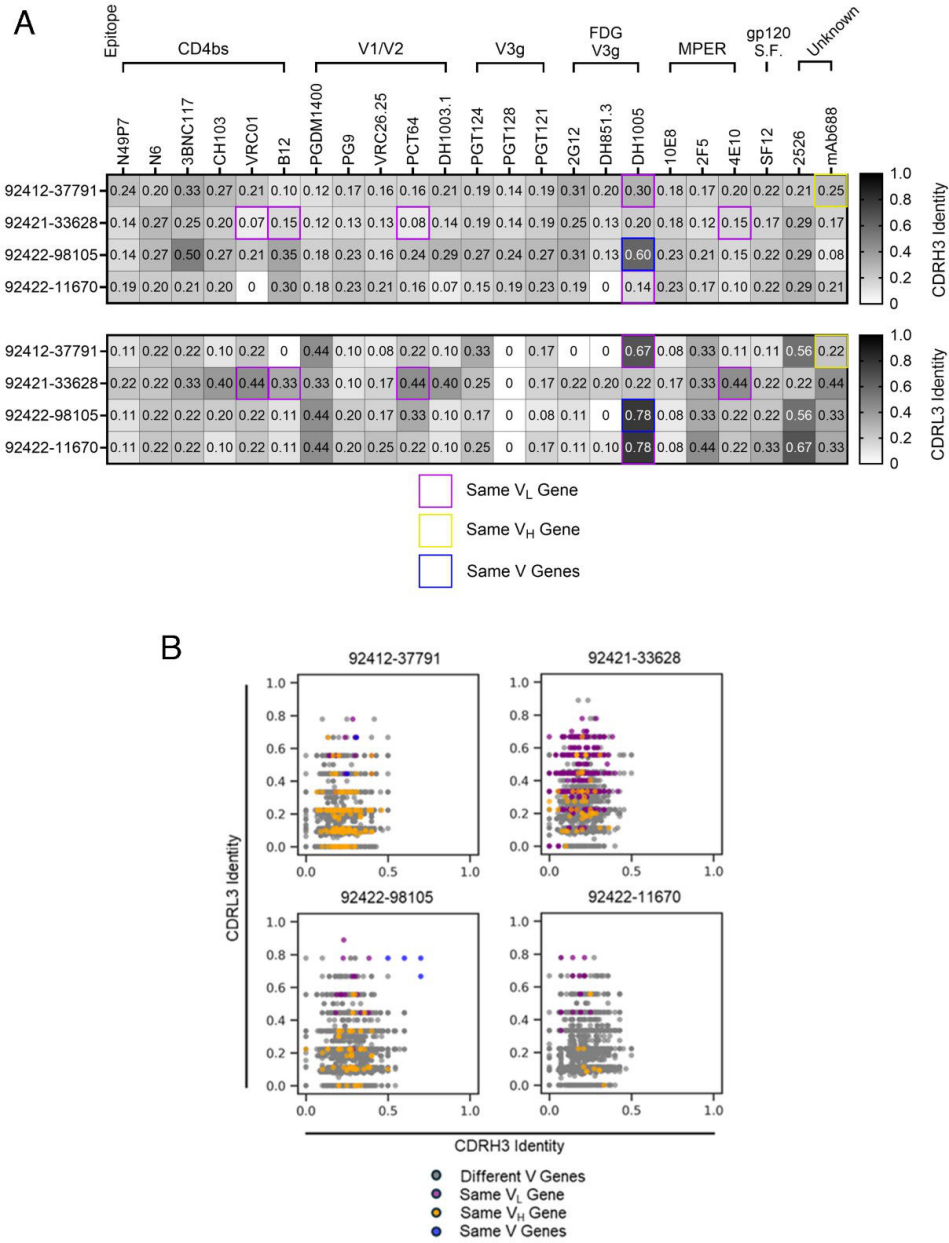
CDRH3 and CDRL3 sequence comparisons. (**A**) HVTN124 mAbs vs. published HIV-reactive bNAbs. The four HVTN 124 mAbs are listed as rows, while the published bNAbs and their known epitopes are shown as columns. The top panel shows comparisons of the CDRH3 amino acid identity, while the bottom panel shows comparisons of the CDRL3 amino acid identity. Both scales range from 0 in white to 1.0 in black, with higher values in black corresponding to higher CDRH3 or CDRL3 sequence identity. Purple boxes signify matching VL genes only, yellow boxes signify matching VH genes only, and blue boxes signify matching both V genes between the respective published bNAbs and the HVTN124 mAbs. Identity refers to the Levenshtein distance based on amino acids, normalized by the length of the longer sequence. (**B**) HVTN124 mAbs vs. publicly available HIV-reactive antibodies. The four HVTN124 mAbs are listed at the top of each graph, with the X-axis listing CDRH3 amino acid identity and the Y-axis listing CDRL3 amino acid identity against publicly available HIV-reactive antibodies. HVTN 124 mAbs were screened against 3,699 unique HIV-reactive mAb sequences (dots) from the SAbDab and PLAbDab public databases. Each colored dot relates to: gray = different V genes, purple = same Vl gene, yellow = same VH gene, and blue = same both V genes. Blue dots were made by aligning against previously published sequences (28, 59). Identity refers to the Levenshtein distance based on amino acids, normalized by the length of the longer sequence.

Sequences of the four HVTN124 mAbs were compared against a panel of known HIV-reactive antibodies, as well as against a panel of publicly available antibody sequences (Fig. 7). Notably, three of the four HVTN124 mAbs exhibited substantial sequence similarity to the previously described glycan-reactive antibody DH1005 (Fig. 7A). Using the amino acid-based Levenshtein distance normalized to the length of the longer sequence, antibodies 92412-37791 and 92422-11670 were found to be encoded by the same light chain germline gene as DH1005, with 67% and 78% identity in the CDRL3 sequences, respectively. Furthermore, antibody 92422-98105 is encoded by the same heavy and light chain germline genes as DH1005, with CDRH3 amino acid identity of 60% and CDRL3 identity of 78%. Together, these results suggest that these HVTN124 mAbs may belong to a public light chain-driven class of glycan-reactive antibodies that can recognize HIV-1.

Next, we compared the four HVTN124 antibodies against a compiled set of 3,699 publicly available HIV-reactive antibody sequences from the SAbDab and PLAbDab databases (Fig. 7B). For each HVTN124 mAb, multiple antibodies were identified in the compiled germline data set to contain matching light chain or matching heavy and light chains with CDRL3 amino acid identities of more than 60%, in some cases reaching 80%–90%. As was the case with the high sequence signature match between 92422-98105 and DH1005, there were multiple antibodies in the compiled data set that were encoded by both the same heavy and light chain germline genes as 92422-98105 and had high CDRH3 and CDRL3 amino acid identities (Fig. 7B). Together, these results suggest that the light chain sequences for the HVTN124 mAbs are commonly found in human antibodies.

### Broadly neutralizing activity toward tier 1 and tier 2 HIV-1 strains

**Fig 8:**
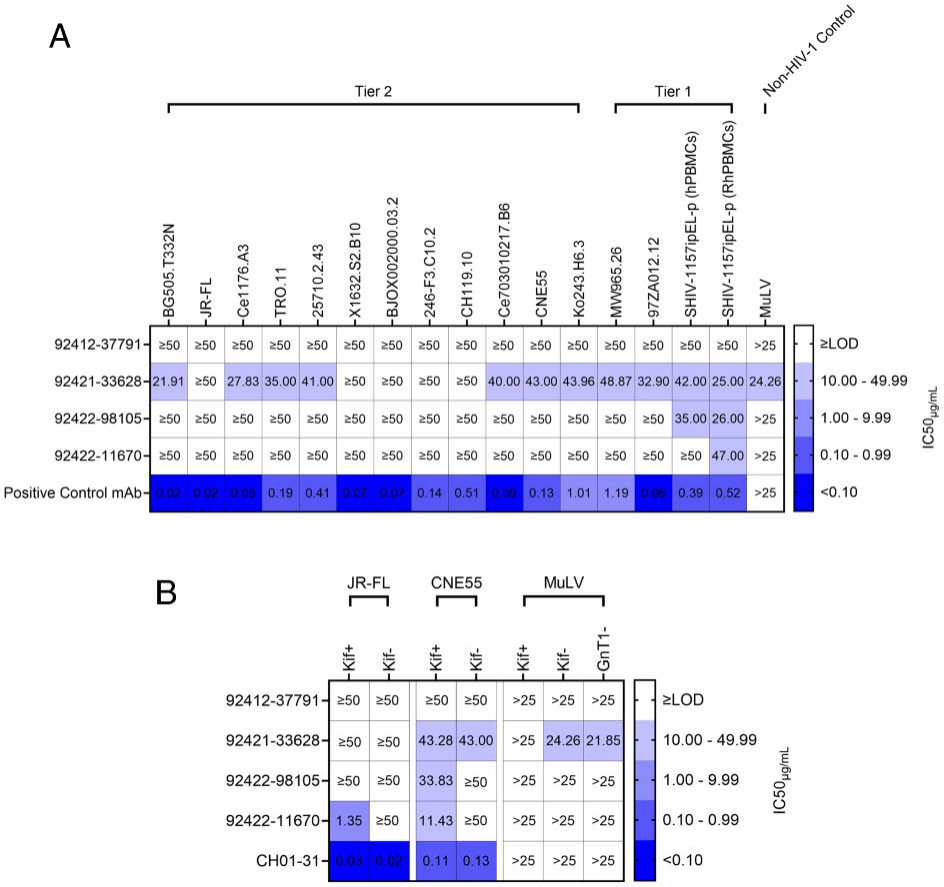
HIV-1 neutralization. (**A**) HVTN124 mAbs vs. HIV-1 pseudovirus and SHIV virus. HVTN 124 mAbs were evaluated for neutralization against pseudotyped HIV-1 from tier 1 and tier 2 strains, which included part of a standardized global representative panel, using a TZM-bl cell neutralization assay. Murine leukemia virus (MuLV) was also included as a non-HIV-1 control. The HVTN124 mAbs are listed as rows, while each HIV-1 pseudovirus strain and MuLV is listed as columns. The positive control mAb CH01-31 was used for all conditions except for BJOX002000.03.2, which used PGT128 as the positive control mAb. For the SHIV virus, VRC01 was used as the positive control mAb. IC_50_ values are listed within each cell as μg/mL mAb, with any values greater than or equal to 50 µg/mL mAb being considered as non-neutralizing against HIV-1 pseudovirus. IC_50_ values greater than 25 µg/mL mAb are considered non-neutralizing against MuLV. Heatmap key on the right is provided to categorize each IC_50_ value into either non-neutralizing in white (≥limit of detection [LOD]), weakly neutralizing in light violet (10.00–49.99), moderately neutralizing in dull lavender (1.00–9.99), potently neutralizing in dark periwinkle (0.10–0.99), and ultra-potently neutralizing in vivid blue (<0.10). (**B**) HVTN124 mAbs vs. HIV-1 pseudovirus containing modified glycans. HVTN124 mAbs were further interrogated for neutralizing activity against pseudotyped HIV-1 from tier 2 strains JR-FL and CNE55, along with MuLV, using a TZM-bl cell neutralization assay. The HVTN124 mAbs and CH01-31-positive control mAb are listed as rows, while the different HIV-1 and MuLV pseudovirus strains are listed as columns. JR-FL and CNE55 pseudoviruses were co-cultured with and without Kifunensine, which is represented as Kif+ or Kif−, respectively. MuLV was also co-cultured with and without Kifuenensine, but was additionally grown in GnT1- HEK293S mammalian cells. IC_50_ values are listed within each cell as μg/mL mAb. The heatmap key, cutoff values, and statistics are the same as in panel (**A**).

Next, we tested whether these antibodies neutralize HIV-1. HIV-1 neutralization was tested against a diverse panel of pseudoviruses, including globally representative HIV-1 strains, additional diverse strains, and PBMC-grown SHIV viruses (60). While three of the four antibodies did not neutralize HIV-1 pseudoviruses, antibody 92421-33628 weakly, yet broadly, neutralized HIV-1 strains belonging to tier 1 and tier 2 (Fig. 8A). Interestingly, 92421-33628 also weakly neutralized murine leukemia virus (MuLV), suggesting an exceptional breadth of neutralization ability. Antibodies 92421-33628, 92422-11670, and 92422-98105 were each able to show weak neutralization when tested against tier 1 R5-tropic SHIV viruses (SHIV-1157ipEL-p) cultured in human PBMCs (hPBMCs) and/or rhesus macaque PBMCs (RhPBMC) (Fig. 8A), which may represent different and more heterogeneous Env glycosylation (61).

Kifunensine is a potent inhibitor of α-mannosidase I, which affects N-glycan processing to only allow cells to produce high-mannose glycans at all N-linked glycosylation sites on HIV-1 Env (62). Because the HVTN 124 mAbs target high-mannose N-glycans, we next tested their ability to neutralize kifunensine-processed HIV-1 pseudovirus. We found that three of the four HVTN124 mAbs were able to neutralize kifunensine-processed HIV-1 pseudovirus, and that kifunensine treatment improved neutralization titers in several cases (Fig. 8B). Most notably, 92422-11670 was unable to neutralize JR-FL without kifunensine treatment (JR-FL Kif-), yet potently neutralized JR-FL with kifunensine treatment (JR-FL Kif+) at ~1 µg/mL half-maximal inhibitory concentration (IC_50_). MuLV with modified glycans was also tested for neutralization, which included kifunensine-processed MuLV and MuLV grown using a N-acetylglucosaminyltransferase-deficient (GnT1-) HEK293S cell line. GnT1- cell lines arrest glycosylation at the Man5 stage, resulting in the similar production of only high-mannose glycans; however, GnT1- pseudoviruses display overall fewer mannose residues than when using kifunensine, giving the glycans of surface antigens of GnT1- pseudoviruses an overall reduced bulkiness (63). Apart from 92421 to 33628, each of the three antibodies showed neutralization against either the Kif+ or GnT1- MuLV, but not both, potentially suggesting somewhat different glycan requirements for recognition by these antibodies. Full percent neutralization curves against all viruses used can be found in Fig. S8A and B. Together, these results show that HIV-1 neutralization by HVTN124 glycan-reactive antibodies can be modulated by virus glycosylation, with antibody 92421-33628 showing broad, albeit less potent, neutralization ability against a wide range of diverse HIV-1 strains, including heterologous tier 2 strains.

### Neutralizing activity against influenza, SARS-CoV-2, and HCV

**Fig 9:**
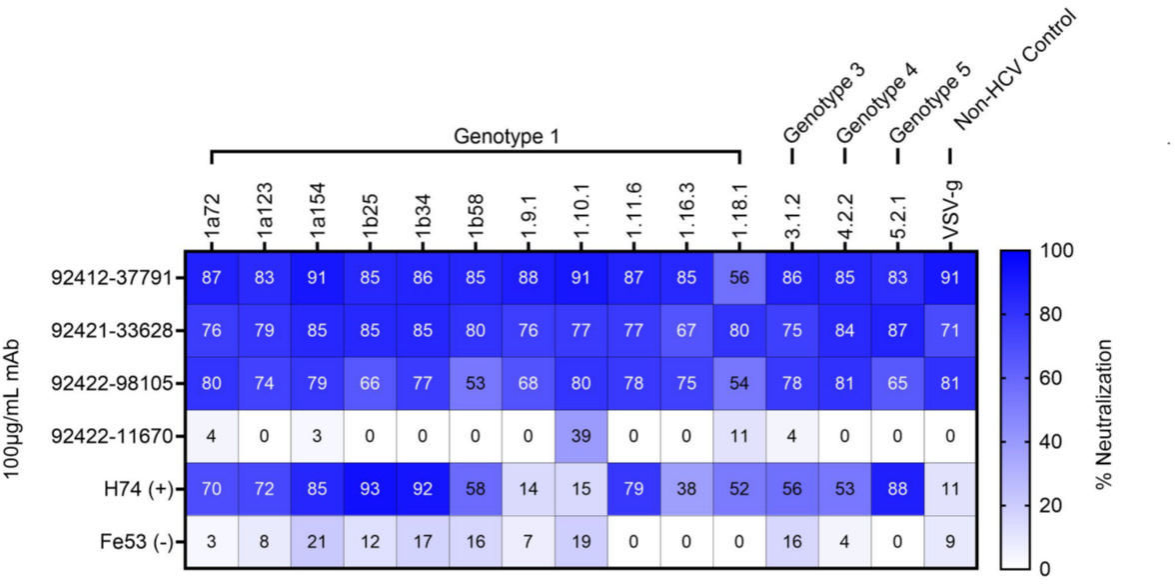
HCV neutralization. HVTN 124 mAbs vs. panel of HCV pseudoparticles. HVTN 124 mAbs were tested for neutralizing activity against a panel of 14 different HCVpps, along with VSV as a non-HCV control condition. The rows list each of the different mAbs used at 100 µg/mL mAb, with H74 and Fe53 being used as positive and negative controls, respectively. The columns list the different viruses and their respective genotypes against which each mAb was tested. Viral tiers for each strain are as follows: tier 1 (5.2.1, 1.11.6), tier 2 (1a154, 1b34, 1a138, 1a123, 1.9.1, 1b25), tier 3 (1.16.3, 4.2.2, 1a72, 1.10.1, 1b58), and tier 4 (1.18.1, 3.1.2). The percent neutralization heatmap key is provided along the X-axis from 0 (white) to 100 (dark blue) percent neutralization.

Since the HVTN124 mAbs recognize antigens from viruses other than HIV-1, we assessed whether these antibodies could also neutralize non-HIV viruses, which included the following: influenza, rVSV-SARS-CoV-2 D614G, rVSV-MERS-CoV, rVSV-SARS-CoV, and HCV. None of the antibodies were found to neutralize any of the tested influenza (Fig. S9) or coronavirus (Fig. S10) strains, suggesting that the HVTN124 mAbs are targeting non-neutralizing epitopes that are not critical for viral entry and fusion. In contrast, strong neutralization activity was observed for three of the four antibodies against 14 HCV pseudoparticles (pps) from multiple genotypes across tiers 1, 2, 3, and 4 (Fig. 9). These three antibodies also neutralized vesicular stomatitis virus (VSV), in agreement with the observed MuLV neutralization in the HIV-1 pseudovirus assay reported above, further supporting that glycan-reactive antibodies can target conserved glycan structures to neutralize viruses with different host and cell tropism. The breadth of the HCVpps used for neutralization included Genotype 1, Genotype 3, Genotype 4, and Genotype 5 strains to account for the broad viral diversity. Together, these results suggest that the HVTN124 glycan-reactive antibodies are capable of neutralizing diverse viruses, illuminating the extraordinary complexity of the immune system to recognize N-linked glycans on viral antigens.

### Antibody Fc effector functions against HIV-1, influenza, and SARS-CoV-2

**Fig 10:**
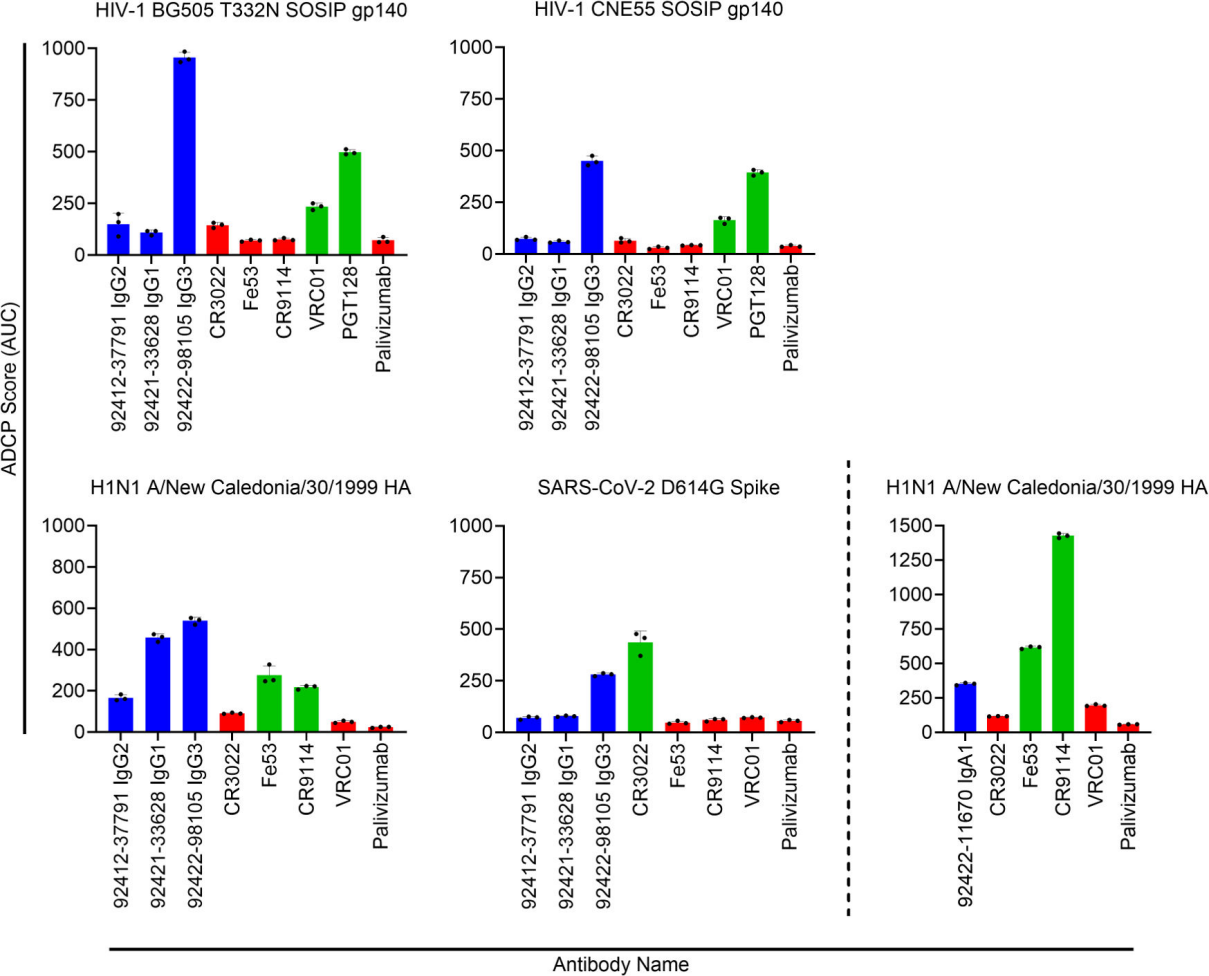
HVTN 124 mAb ADCP activity. HVTN 124 ADCP activity using native isotypes. ADCP activity was measured against HIV-1 BG505 T332N SOSIP gp140, HIV-1 CNE55 SOSIP gp140, influenza H1N1 A/New Caledonia/30/1999 HA, and SARS-CoV-2 D614G Spike. Antigens were each biotinylated and conjugated to fluorescent neutravidin beads, then mixed with each mAb. Antibodies are listed along the X-axis, while the Y-axis lists the ADCP score as AUC. The control mAbs used include the following: VRC01, PGT128 = HIV-1; Fe53, CR9114 = influenza; CR3022 = SARS-CoV-2. Palivizumab was included as a negative control antibody. Antibodies are color-coded by HVTN124 mAbs (blue), positive control mAbs (green), and negative control mAbs (red). For IgG isotypes, THP-1 monocytes were subsequently added, and ADCP was calculated based on the engulfment of the antigen-coated beads by the THP-1 cells as measured by flow cytometry. For IgA isotypes, DMSO-differentiated HL60 cells were subsequently added, and ADCP was calculated based on the engulfment of the antigen-coated beads by the THP-1 cells as measured by flow cytometry. Antigen engulfment is indicated as an ADCP score. All experiments were performed in triplicate. Additional plots for the native IgA and non-native isotypes can be found in Fig. S11A and B.

In addition to virus neutralization, antibodies can also mediate immune responses through their Fc domain. After engaging with Fc gamma receptors (FcγRs) on effector cells, antibodies can trigger mechanisms such as antibody-dependent cellular cytotoxicity (ADCC) and/or antibody-dependent cellular phagocytosis (ADCP). Both ADCC and ADCP are two critical immune mechanisms that can aid in eliminating infected cells. The ability to elicit antibodies that can trigger ADCC and/or ADCP is of interest for HIV-1 vaccine design to provide an additional mechanism of controlling HIV-1 infection outside of neutralization. ADCP activity of the four HVTN124 glycan-reactive antibodies was tested against HIV-1 BG505 T332N SOSIP gp140, HIV-1 CNE55 SOSIP gp140, influenza H1N1 A/New Caledonia/30/1999 HA, and SARS-CoV-2 D614G spike (Fig. 10). When tested using the non-native isotypes, we observed weak ADCP activity against H1N1 A/New Caledonia/30/1999 HA with 92422-11670, along with weak ADCP activity against HIV-1 BG505 N332T SOSIP gp140 and HIV-1 CNE55 SOSIP gp140 with 92412-37791 and 92422-98105 (Fig. S11B). However, when native isotypes were used, ADCP activity noticeably increased against each antigen (Fig. 10). Most significantly, the native IgG3 isotype of 92422-98105 showed stronger ADCP activity compared to the positive controls VRC01 and PGT128 against HIV-1 BG505 N332T SOSIP gp140 and HIV-1 CNE55 SOSIP gp140, while also showing strong ADCP activity against H1N1 A/New Caledonia/30/1999 HA and SARS-CoV-2 D614G. Also of note, the native IgG1 isotype of 92421-33628 showed similar ADCP activity to 92422-98105 IgG3 against H1N1 A/New Caledonia/30/1999 HA, although it did not yield a positive ADCP activity score against the other antigens. As expected, the native IgG2 isotype for 92412-37791 did not show ADCP activity, with it being reported that the IgG2 subclass is a poor inducer of effector functions (64). The native IgA1 isotype for 92422-11670 was also found to only show strong ADCP activity against H1N1 A/New Caledonia/30/1999 HA, which could be due to pre-existing immunity against flu from repeated infections or vaccinations. ADCC activity of the four HVTN124 glycan-reactive antibodies, using the non-native isotypes, was tested against HIV-1 BG505 T332N SOSIP gp140, influenza H1N1 A/New Caledonia/30/1999 HA, and SARS-CoV-2 D614G spike (Fig. S12). We observed that for all three antigens, antibodies 92412-37791 IgG1 and 92422-11670 IgG1 displayed the strongest ADCC activity comparable to the positive controls (Fig. S12). Together, these results show that all four HVTN124 glycan-reactive antibodies can mediate Fc effector functions, albeit with differing strengths in ADCP and ADCC responses.

## DISCUSSION

Antibody responses to HIV-1 infection have been extensively studied, resulting in the identification of a wide range of broadly neutralizing antibodies, new insights into the genetic and structural features associated with effective protection against infection, and the discovery of new sites of virus vulnerability as potential templates for antibody-based vaccines. In contrast, there is still limited understanding of antibody responses to HIV-1 vaccination in humans. Similarly, mapping the role of pre-existing antibody repertoires on modulating responses to HIV-1 vaccination has not yet been performed; rather, efforts to date have extensively focused on identifying rare precursors for difficult-to-elicit broadly neutralizing antibodies (65). The antibody repertoire in HIV-naive humans is highly complex, comprising antibodies with diverse sequence features and phenotypes that have the potential to engage with the vaccine antigens upon immunization.

Recent work suggests that one such class of antibodies that have the capability of engaging HIV-1 vaccine antigens in HIV-naïve individuals are glycan-reactive FDG antibodies (28). Multiple efforts have previously described how glycan-reactive antibodies uniquely display broad reactivity across multiple viral families (28, 66, 67). As such, FDGs (and, more generally, glycan-reactive) antibodies may represent an immune system mechanism that provides a first line of defense against a broad range of viral pathogens in humans. At the same time, this class of antibodies may also be among the first to engage with a vaccine antigen upon immunization, highlighting the importance of deciphering the features and functional phenotypes associated with these antibodies.

In this report, we discovered and characterized a set of glycan-reactive antibodies isolated from participants in the HVTN124 HIV-1 vaccine trial. While these antibodies exhibited stronger binding to the trimeric Env-derived SOSIP antigens, binding to the gp120 HVTN124 vaccine antigens was also observed. Notably, for three of the four antibodies, reactivity against the HIV-1 antigens was not observed when reverted to germline. Thus, mutations acquired (perhaps in germinal centers amongst bystander B cells) may have enabled *de novo* vaccine antigen recognition, perhaps driving further vaccine antigen-based selection and affinity maturation. Other factors, such as BCR vs. monoclonal antibody affinity and potential differences in true vs. inferred germline precursor sequences, could also play a role, thus preventing any definitive causal conclusions about the generation of these antibodies.

We also determined that the HVTN 124 antibodies are weakly autoreactive against intracellular and extracellular targets in some but not all assays. Autoreactive bNAbs such as 2F5 and 2G12 have been previously reported to target cellular components such as the nucleus and cytoplasm (28). Despite this autoreactivity, prior studies have shown that autoantibodies do not artificially skew virus neutralization results in cell-based assays, including those performed in TZM-bl and MDCK cells (28, 68, 69). These standardized assays incorporate cell- and virus-only controls to monitor cytotoxicity or non-specific inhibition, helping ensure that measured neutralization is specific to virus-antibody interactions. Given that these antibodies show antiviral functional activity, vaccine regimens will have to consider the balance between marginal autoreactivity and broad antiviral protection, with additional consideration given to isotype skewing in the response, which, in turn, would impact Fc receptor and complement engagement, as these could differentially impact the likelihood of host autoimmune pathology.

Among the four isolated antibodies, 92421-33628 appeared to have the most intriguing and unique profile. This antibody showed an ability to neutralize, albeit weakly, a diverse set of HIV-1 viruses from tiers 1, 2, and 3 when tested in a pseudovirus assay. To our knowledge, this is the first report of a glycan-reactive broadly neutralizing antibody that was not obtained from an HIV infection sample, demonstrating that these types of antibodies can be elicited outside of an HIV-1 infection. Interestingly, and in line with prior studies, three of the antibodies showed neutralization ability against kifunensine-treated pseudovirus, suggesting that the type of glycans present on the surface of the virus can modulate neutralization by these types of glycan-reactive antibodies (28). Importantly, these three antibodies also exhibited neutralization of SHIV viruses cultured from PBMCs, which represent different and potentially more heterogeneous glycosylation compared to the HIV-1 pseudovirus produced in cell lines. Furthermore, one of these neutralizing antibodies (92422-98105) showed high sequence similarity to a previously published glycan-reactive antibody in both the heavy and light chain sequences, as part of a public class of glycan HIV-reactive antibodies.

To fully understand the protective potential of vaccine-elicited antibodies, it is critical to not only consider their specificity and affinity, but also their isotype, which can influence Fc-mediated effector functions. It has previously been reported that the longer and more flexible hinge region of IgG3 antibodies allows this isotype to more effectively engage with Fc gamma receptors (FcγRs) on phagocytes, thereby enhancing ADCP activity (70, 71). HIV-1 vaccines aiming to skew the antibody repertoire toward preferentially engaging with IgG3 antibodies can help further improve viral control in a natural infection.

Together, the results reported here build on current knowledge about the features and functional phenotypes of glycan-reactive antibodies and their potential role as a component of effectively responding to HIV-1 vaccination in HIV-naïve repertoires. These efforts motivate further research toward mechanistically understanding the molecular and developmental basis of these glycan-reactive antibodies, along with how this class of antibodies can be better targeted for large-scale engagement in future HIV-1 vaccine trials.

## MATERIALS AND METHODS

### HVTN124 study design and participants

HVTN124 was a Phase 1 clinical trial with a randomized, double-blind, placebo-controlled design that aimed to assess both the safety profile and the immunological response induced by an adjuvanted polyvalent HIV-1 vaccine containing DNA plasmids from four different clades, in addition to four adjuvanted gp120 proteins. The trial was implemented at six different sites in the United States: Case Western Reserve University (Ohio), Emory University (Georgia), Fenway Community Health Center (Massachusetts), University of Alabama at Birmingham (Alabama), University of Pennsylvania (Pennsylvania), and University of Rochester (New York). The first part of the trial focused on determining the safety properties of the protein-GLA-SE formulation. The second part measured the immune responses generated by prime-boost and coadministration approaches, while ensuring continued safety measures. The research protocol, made publicly available, was approved by the biosafety and ethics review committee of each institution involved. Participants were recruited through print, online, and in-person outreach efforts. Eligibility criteria included being HIV-negative, 18–50 years old, having minimal risk for HIV-1 exposure, having no major medical conditions, not taking systemic immunosuppressive drugs, and meeting laboratory screening criteria. Gender was self-reported with inclusive response options. Written informed consent was obtained from all participants before any study procedures were started.

### Immunogen panel expression, purification, and validation

HIV-1 immunogens were designed using the SOSIP platform to yield soluble Env proteins that are stabilized in the pre-fusion conformation. SOSIP proteins include the following mutations: an intermolecular disulfide bond between gp120 and gp41 (A501C and T605C), a trimer-stabilizing mutation (I559P), a truncated gp41 transmembrane region at position 664, an I201C/A433C mutation to inhibit CD4-induced movement of Env, and a variable flexible serine-glycine linker between gp120 and gp41 (positions 507 and 512) to create single-chain constructs (30). SOSIP immunogens were transiently transfected into Expi293F cells in FreeStyle F17 expression media (Thermo Fisher). Supplements were added at a final concentration of 0.1% Pluronic Acid F-68 and 1× Glutamax. Cells were transfected using the Expifectamine transfection reagent (Thermo Fisher Scientific), followed by culturing in a shaker for 5–7 days at 8% CO_2_ at 37°C. Following transfection, cell cultures were centrifuged at 4,000 × *g* for 15 minutes to form a stable cell pellet. Centrifuged cell culture supernatant was then filtered using Nalgene Rapid Flow Disposable Filter Units with PES membrane (0.45 µM), then run slowly over a chromatography column with agarose-bound Galanthus nivalis lectin (Vector Laboratories cat no. AL-1243-5) at 4°C. After the supernatant had flowed through, the column was washed with 1× phosphate-buffered saline (PBS), then the immunogen was eluted with 30 mL of 1 M methyl α-D-mannopyranoside. The eluted immunogen was then buffer exchanged in 1× PBS three times, followed by concentrating using 100 kDa Amicon Ultra centrifugal filter units. The concentrated immunogen was run through a Superdex 200 Increase 10/300 Gl on the AKTA FPLC system. Any peaks indicative of Env trimers were collected based on elution volume, followed by validation of molecular weight using SDS-PAGE gel electrophoresis and antigenicity using an ELISA format against Env-specific antibodies.

### Antigen biotinylation

All immunogens used for LIBRA-seq were non-specifically biotinylated using the EZ-Link Sulfo-NHS-Biotinylation Kit (Thermo Scientific cat no. 21425) according to the manufacturer’s protocol.

### Oligonucleotide barcodes and conjugation to antigens

Oligonucleotides used in the LIBRA-seq pipeline contain a 15 base pair barcode that is unique for each antigen in the panel. During sample processing with the 10× Genomics platform, a part of the oligonucleotide sequence will anneal to the 10× Genomics bead, specifically at the template switch oligonucleotide. Lastly, each oligonucleotide contains a truncated TruSeq small RNA sequence as follows: 5′-CCTTGGCACCCGAGAATTCCANNNNNNNNNNNNNNNCCCATATAAGA*A*A-3′.

N’s in this example represent the unique barcode for distinguishing each antigen. Oligonucleotides were ordered from Sigma-Aldrich and IDT with a 5′ amino modification and were HPLC purified. During LIBRA-seq analysis, the following unique barcodes were used with their respective antigens:

HVTN124 Clade A gp120 (GCTCCTTTACACGTA), BG505 N332T SOSIP gp140 (CAGATGATCCACCAT), KNH1144 SOSIP gp140 (ACAATTTGTCTGCGA), HVTN124 Clade B gp120 (AGACTAATAGCTGAC), B41 SOSIP gp140 (TCACAGTTCCTTGGA), TRO.11 SOSIP gp140 (AACCTTCCGTCTAAG), HVTN124 Clade C gp120 (TACGCCTATAACTTG), CZA97 N332T SOSIP gp140 (TAACTCAGGGCCTAT), ZM106.9 SOSIP gp140 (CAGCCCACTGCAATA), HVTN124 Clade AE gp120 (CTTCACTCTGTCAGG), CNE55 SOSIP gp140 (TCCTTTCCTGATAGG), influenza H1N1 A/New Caledonia/30/1999 HA (AATCACGGTCCTTGT)

Each unique oligonucleotide barcode was directly conjugated to its respective antigen using the Solulink Protein-Oligonucleotide Conjugation kit (TriLink, S-9011) according to the manufacturer’s protocol. The antigen and oligonucleotide were first desalted, then the oligonucleotide was modified with a 4FB crosslinker while the biotinylated antigen was separately modified with S-HyNic. These two products were then mixed and incubated to allow a stable bond to form between the antigen and oligonucleotide. Concentrations of the antigen-oligonucleotide conjugates were determined using a BCA assay, followed by measuring the S-HyNic and 4FB molar substitution ratios of each conjugate using the NanoDrop based on the Solulink protocol. AKTA FPLC was then used to remove excess oligonucleotide from the antigen-oligonucleotide conjugates to reduce noise during Illumina Next-Generation Sequencing by VANTAGE, with the removal of excess oligonucleotide being validated using SDS-PAGE silver stains.

### 10× Genomics single-cell processing for Illumina next-generation sequencing

Following single-cell sorting, samples were loaded onto a Chromium Controller microfluidics device (10× Genomics) and processed as previously described above. A target capture of 10,000 B cells was used based on the recommended specifications for the 10× Genomics platform.

### Bioinformatics analysis

By utilizing our lab’s established R Studio pipeline, the paired-end FASTQ files from the oligonucleotide libraries were input to first process and label cell barcode reads. This initial step results in a matrix of unique molecular identifiers (UMIs) and antigen barcodes. Next, files related to BCR contigs were processed using CellRanger 3.1.0 (10× Genomics) and GRCh38 V(D)J 7.0.0 as a reference, with the antigen barcode libraries processed in the earlier step also being processed using CellRanger (10× Genomics). Any cell barcodes that overlapped with the antigen barcodes and UMIs were then subject to further analysis. Cell barcodes were eliminated from the matrix if they contained non-functional heavy and/or light chain sequences, in addition to if the cell barcodes were associated with multiple functional heavy and/or light chain sequences, with both criteria aiming to eliminate any multiplets. BCR contigs (file output by CellRanger as filtered_contigs.fasta) were aligned to IMGT reference genes using HighV-Quest. This output was analyzed using ChangeO, then combined with the antigen barcode UMI score matrix. The LIBRA-seq score (LSS) for each antigen in the panel against every B cell was then calculated as previously described (29).

### Enzyme-linked immunosorbent assay

Enzyme-linked immunosorbent assay (ELISA) for immunogens was done in an Immulon 2 HB 96-Well Microtiter EIA Plate. Immunogens were diluted to 2 µg/mL in 1% BSA in PBS-T, then added to the plates and incubated overnight at 4°C. The next day, plates were washed using 0.05% Tween 20 (PBS-T) to remove any unbound antigen, then blocked in 5% BSA in PBS-T for 2 hours at room temperature. After washing again after incubating, the antibody dilution series in 1% BSA in PBS-T were plated and incubated for 1 hour at room temperature, then washed. The secondary antibody, goat anti-human IgG conjugated to peroxidase, was applied to each well at a 1:10,000 dilution in 1% BSA in PBS-T, then incubated for 1 hour at room temperature. After washing again, the plates were developed by adding TMB substrate to each well for 5 minutes at room temperature. The reaction was quenched by adding 1N sulfuric acid, where plates were then read at 450 nm. Values were then subtracted from the average A450 nm values from the no-mAb condition to account for background noise. Concentrations were then transformed, where µg/mL mAb was converted to ng/mL mAb, and the log10 of ng/mL mAb was taken to give a new concentration as log[mAb] (ng/mL). Area under the curve was then taken, where the baseline was set to Y = 0, the minimum peak height was set to ignore peaks that are less than 10% of the distance from minimum to maximum Y, and peak direction was defined as having peaks that went above baseline.

### Mannose-competition ELISA

Mannose competition ELISAs were performed as described above, but with minor modifications. Following coating with CNE55, plates were blocked for 1 hour at room temperature in either 1M D-(+)-mannose 5% BSA PBS-T or 5% BSA PBS-T. Primary antibodies were prepared in either 1M D-(+)-mannose 1% BSA PBS-T or 1% BSA PBS-T, starting at 10 µg/mL with a fivefold serial dilution. After washing three times, primary antibodies were added to each plate for 1 hour at room temperature. Following another wash step, goat anti-human IgG conjugated to peroxidase was added at a 1:10,000 dilution in 1% BSA PBS-T for 1 hour at room temperature. After washing again, the plates were developed and quenched as described above. Values were also transformed as described previously.

### Enzymatic deglycosylation of HIV-1 CNE55 SOSIP gp140

The glycan-dependency of antibodies was evaluated by treating the antigen under different conditions, then testing by ELISA. Antigen under the native condition was quickly thawed after being taken from –80°C storage and had no enzymatic treatment applied. The 37°C condition had the antigen diluted in 1× Glycobuffer 2, then incubated at 37°C overnight. The 37°C + PNGase F condition had the antigen diluted in 1× Glycobuffer 2 and incubated with PNGase F at 37°C overnight. The 37°C + Endo H condition had the antigen diluted in 1× Glycobuffer 2 and incubated with Endo H at 37°C overnight. The 37°C + O Glycosidase condition had the antigen diluted in 1× Glycobuffer 2 and incubated with O Glycosidase at 37°C overnight. The 95°C condition had the antigen diluted in Glycoprotein Denaturing Buffer and boiled at 95°C for 10 minutes, then chilled on ice for 2 minutes. 1 µL of enzyme was used per 10 µg of total antigen for each condition. The different antigen conditions were then directly coated onto ELISA plates and assayed as previously described in the ELISA methods section. Values were also transformed as described previously. To normalize values against the native condition, values gathered from calculating the AUC were applied to the normalization analysis option in GraphPad Prism. Settings used included setting 0% defined as Y = 0, 100% defined as the AUC value from the native condition for each separate antibody, and presenting the results as percentages. This resulted in the native condition being set at 100% for all antibodies across the different conditions.

### Glycan microarray

Antibodies were tested against a panel of 562 unique glycan structures following a previously reported microwave-assisted wet-erase protocol, but with several exceptions (50). PBS was used as a buffer instead of TSM; therefore, the binding buffer was PBS with 1% BSA. Antibodies were diluted in binding buffer to 50 µg/mL. The first detection step used anti-human IgG-biotin at 5 µg/mL. The second detection step used streptavidin-Cy5 at 0.5 µg/mL. The remaining washing and scanning steps followed the published method mentioned previously. Figures of glycan structures were gathered using GlycoGlyph (49).

### AtheNA autoantigen panel

Monoclonal antibody reactivity was measured using the AtheNA Multi-Lyte ANA-II Plus test kit (Zeus Scientific, Inc.), which tested against nine autoantigens (SSA/Ro, SS-B/La, Sm, ribonucleoprotein (RNP), Scl 70, Jo-1, dsDNA, centromere B, and histone). Antibodies were incubated with AtheNA beads for 30 minutes at concentrations of 50, 25, 12.5, or 6.25 µg/mL mAb. After washing beads, they were incubated with secondary and read on the Luminex platform as specified in the kit protocol. AtheNA software was used to analyze the data. Positive samples were those that received a score >120, while negative samples received a score of <100. Values between 100 and 120 were considered intermediate.

### Cardiolipin reactivity

Monoclonal antibody reactivity to cardiolipin was measured using the Quanta Lite ACA IgG III kit (Inova Diagnostics Inc.) by diluting antibodies to final concentrations of 100, 50, 25, and 12.5 µg/mL in sample diluent. 100 µL of each sample was added to assay plates along with kit controls, and the kit SOP was followed for the remainder of the assay. Phospholipid units (GPL) were calculated against a linear standard curve per kit instructions. An individual well was positive if GPL was >20, negative if <15 and indeterminate if between 15 and 20. Any given antibody needed to be positive for two consecutive wells (i.e., to at least 50 µg/mL) to be considered cardiolipin positive.

### Cell monolayer immunofluorescence

Monoclonal antibody binding to whole, permeabilized, uninfected HEp-2, HEK293T, TZM-bl, Jurkat, MDCK, and HEK293F cell monolayer cultures was performed via indirect immunofluorescence. In all, 25,000 pelleted cells were resuspended in 4% formaldehyde, methanol-free (Cell Signaling Technology #47746), then plated directly to black 96-well microscopy plates (Ibidi #89606) for 20 minutes at room temperature. Cells were then gently permeabilized using DPBS containing 0.1% Triton X-100 for 10 minutes at room temperature. Potential non-specific interactions were blocked using DPBS containing 5% BSA for 2 hours at room temperature. Primary antibodies were added at 25 µg/mL for 30 minutes at room temperature. A 1:500 dilution of goat anti-human IgG-AF488 (Southern Biotech #2040-30) was applied to cells for 30 minutes at room temperature in the dark. A 1:5 dilution of NucBlue DAPI (Thermo Fisher Scientific #R37606) was applied to cells to counterstain for 15 minutes at room temperature in the dark. Cells were gently washed three times for 5 minutes each with DPBS containing 0.1% Tween-20 between each step. Primary and secondary antibodies were resuspended in DPBS containing 1% BSA and 0.1% Tween-20. Cells were resuspended in DPBS and imaged using the Zeiss LSM 880 Airyscan Confocal Microscope at 40× magnification with the LD C-Apochromat 40×/1.1 W Korr objective. AF488 channel was imaged at 1 Airy Unit at 2% laser intensity, with the master gain being set to 650, digital offset set to 10, and digital gain set to 2.5. The DAPI channel was imaged at 1.25 Airy Unit at 2% laser intensity, with the master gain being set to 450, digital offset set to 2, and digital gain set to 1. Images were gathered with an 8-bit depth and a scan time of 10 seconds with 4× line averaging to reduce noise. Figures were generated using Zeiss Zen 3.11.

### Intracellular and extracellular flow cytometric analysis

HEp-2, HEK293T, TZM-bl, Jurkat, and MDCK cell lines were harvested for flow cytometry staining. Cells were incubated with Alexa Fluor 700 NHS Ester (Succinimidyl Ester) (Thermo Fisher Scientific) prior to fixation/permeabilization to enable viability assessment. For extracellular staining, cells were incubated on ice with 25 µg/mL unlabeled experimental and control IgG mAbs, or flow cytometry staining buffer alone (secondary controls). Cells were washed, and rat anti-human IgG Fc-PE/Cy7 (1:1500, clone M1310G05) was used to detect human IgG mAbs. For intracellular staining, cells were fixed and permeabilized using the BD Cytofix/Cytoperm kit, followed by incubation with 25 µg/mL experimental or control mAbs. Cells were washed, and rat anti-human IgG Fc-PE/Cy7 (1:1500, clone M1310G05) was used to detect human IgG mAbs. All cells were resuspended in 1% paraformaldehyde (PFA) in 1× PBS and stored at 4°C prior to acquisition on a BD Biosciences LSR Fortessa flow cytometer. Data were analyzed using FlowJo software (BD Biosciences). Gating strategies and IgG + plots can be found in Fig. S6A through E.

### NSEM

Antibodies from −80°C were separately thawed at RT in an Al block for 5 min. Samples were then diluted to 20 µg/mL with 0.02 g/dL Ruthenium Red in HBS (20 mM HEPES, 150 mM NaCl, pH 7.4) buffer. After 10–15 minute incubation, samples were applied to a glow-discharged carbon-coated EM grid for 8–10 seconds, blotted, consecutively rinsed with 2 drops of 1/20× HBS, and stained with 2 g/dL uranyl formate for 1 minute, blotted, and air-dried. Grids were examined on a Philips EM420 electron microscope operating at 120 kV and nominal magnification of 49,000×, and 20 images were collected on a 76 Mpix CCD camera at 2.4 Å/pixel. Images were analyzed by 2D class averages using standard protocols with Relion 3.0 (72).

### Ramos cell line

The pipeline for generating Ramos cell lines described here can be found in a previous publication by Weaver et al. (73).

### Published and public antibody analysis

The published and public reference libraries use paired heavy chain and light chain human BCR sequences from published sources, and the SAbDab and PLAbDab public databases, respectively. We used custom Python scripts utilizing NumPy and Pandas to determine published and public clones between our data and a reference library. The identification of published and public clonotype similarity utilized a cutoff of 70% amino acid sequence identity for the CDRH3 and CDRL3 regions, in addition to containing matching heavy variable (V_H_), light variable (V_L_), and joining (J) gene usage. Levenshtein distance was used for comparison in CDR3 identities. When comparing CDRH3 and CDRL3 of different lengths, gaps are penalized the same as mismatches.

### HIV-1 and MuLV pseudovirus and SHIV virus production and neutralization assays

HIV-1 and MuLV pseudoviruses were produced by co-transfecting HEK293T cells or GlcNAc transferase I enzyme-deficient 293S cells (293S/GnTI-) with an Env-expressing plasmid and an Env-deficient HIV-1 backbone vector pSG3ΔEnv (BEI #11051) as previously reported (74). SHIV virus (SHIV-1157ipEL-p) was cultured using CD8-depleted human or rhesus PBMCs as previously reported (61). The optimal tissue culture infectious dose of each pseudovirus or SHIV was determined by titrating in TZM-bl cells. Antibody neutralization was further characterized using the TZM-bl cell-based assay (68). This standardized assay quantifies the inhibition of TZM-bl cell infection by Env-pseudotyped viruses through antibody-mediated activity. The panel of viruses included tier 1 and tier 2 strains, a murine leukemia virus (MuLV), and PBMC-grown SHIV viruses. Virus neutralization was measured as a function of reductions in luciferase (Luc) reporter gene expression after a single round of infection in TZM-bl cells. Briefly, a pre-titrated dose of virus was incubated with serially diluted antibodies in duplicate for 1 hour at 37°C in 96-well plates, followed by the addition of freshly trypsinized TZM-bl cells containing DEAE-Dextran. Assay plates are incubated at 37°C for 44–72 hours, allowing a single round of infection in TZM-bl cells. Results are displayed as the antibody concentration at which relative luminescence unit (RLU) is reduced to 50% (IC_50_) compared to virus control wells.

### H1N1 and H3N2 influenza virus production and neutralization assay

Production of replication-restricted reporter (R3) H1N1 and H3N2 influenza viruses was described previously. Viral genomic RNA encoding functional PB1 (R3ΔPB1 viruses) was replaced with a gene encoding a fluorescent protein. R3ΔPB1 viruses were rescued and propagated in cell lines stably expressing PB1 via reverse genetics. Viral stocks were titered by determining the number of fluorescent units per mL (FU/mL). Each mAb was diluted in Opti-MEM to 100 µg/mL, serially diluted, mixed 1:1 with viruses, and allowed to co-incubate for 1 hour in a humidified 37°C incubator (5% CO_2_). Viruses were diluted to 1 × 10^4^ to 4 × 10^4^ FU/mL in Opti-MEM prior to mixing. MDCK-SIAT1 cells (Millipore Sigma #05071502) expressing PB1 were then harvested, washed in Opti-MEM, diluted to 1 × 10^6^ cells/mL, added to the mAb-virus mixtures (3:1 mAb-virus to cells), then immediately transferred to a 384-well plate format in quadruplicate. Plates were then incubated for 18–22 hours at 37°C in a humidified incubator (5% CO_2_). After incubation, the fluorescent units of each well were counted using a Celigo Image Cytometer (Nexcelom) with a customized red filter to detect mKate2/TdKatushka2 reporter signals. The percent neutralization was calculated by constraining the virus control (virus + cells) as 0% and the cell control (cells only) as 100% and plotted against mAb concentration.

### rVSV-SARS-CoV-2, rVSV-MERS-CoV, and rVSV-SARS-CoV virus production

The generation of a replication-competent VSV expressing S proteins of respective CoVs that replaces the VSV G protein was described previously (75). The S protein-expressing VSV viruses were propagated in either Vero-E6 or MA104 cell culture monolayers (African green monkey, ATCC CRL-2378.1) as described previously, and viral stocks were titrated on Vero E6 cell monolayer cultures (75). VSV plaques were visualized using neutral red staining.

### rVSV-SARS-CoV-2, rVSV-MERS-CoV, and rVSV-SARS-CoV neutralization using real-time cell analysis

To determine the neutralizing activity of IgGs, we used a real-time cell analysis (RTCA) assay on an xCELLigence RTCA MP Analyzer (ACEA Biosciences Inc.) that measures virus-induced cytopathic effect (CPE) as previously described (76). Briefly, 50 µL of cell culture medium (DMEM supplemented with 2% FBS) was added to each well of a 96-well E-plate using a ViaFlo 384 liquid handler (Integra Biosciences) to obtain background reading. A suspension of 18,000 Vero-E6 cells in 50 µL of cell culture medium was seeded in each well, and the plate was placed on the analyzer. Measurements were taken automatically every 15 minutes, and the sensograms were visualized using RTCA software version 2.1.0 (ACEA Biosciences Inc). Respective VSV-CoV (0.05 MOI, 150 PFU per well) was mixed 1:1 with a dilution of mAb in a total volume of 100 µL using DMEM supplemented with 2% FBS and incubated for 1 hour at 37°C in 5% CO2. At 16–18 hours after seeding the cells, the virus-mAb mixtures were added in triplicates to the cells in 96-well E-plates. Triplicate wells containing virus only (for maximal CPE in the absence of mAb) and wells containing only Vero cells in medium (for no-CPE wells) were included as controls.

Plates were measured continuously (every 15 minutes) for 48–72 hours to assess virus neutralization. Normalized cell index (CI) values at the endpoint (48–72 hours after incubation with the virus) were determined using the RTCA software version 2.1.0 (ACEA Biosciences Inc.). Results were expressed as percent relative infection in the presence of respective mAb, relative to control wells with no CPE minus CI values from control wells with maximum CPE. IC50 values were determined by nonlinear regression analysis using Prism software.

### HCV pseudoparticle (HCV_PP_) production and neutralization assay

A panel of 14 HCVpps from genotype 1 (UKNP1.11.6, UKNP 1A154, UKNP1B34, UKNP1.9.1, UKNP1A123, UKNP1B25, UKNP1.16.3, UKNP1A72, UKNP1.10.1, UKNP1B58, and UKNP1.18.1), genotype 3 (UKNP3.1.2), genotype 4 (UKNP4.2.2), and genotype 5 (UKNP5.2.1) was produced via lipofectamine-mediated transfection of pNL4-3.Luc.R-E, HCV E1E2, and pAdVantage plasmids into HEK293T cells (77, 78). VSV was included as an HCV-specific control. For testing neutralization, 96-well solid flat white bottom polystyrene TC-treated microplates had 8,000 Hep3B cells applied per well, then incubated overnight at 37°C. To test each mAb, HCVpps were co-incubated with 100 µg/mL mAb for 1 hour, then added in duplicate to Hep3B target cells for 5 hours. Following incubation, the medium was changed to 100 µL of phenol-free Hep3B media, then incubated for 72 hours at 37°C. Infectivity was later quantified using a luciferase-based assay measured in RLUs in Berthold Luminometer (Berthold Technologies Centro LB960). Percent neutralization for each mAb was calculated as [1 − (RLUmAb/ RLUIgG)] × 100]. Percent neutralization for the dilution curves was calculated as [1 − (RLUmAb/RLUPBS)] × 100. H74 and Fe53 were included as positive and negative controls, respectively.

### ADCP assay against native HVTN124 antibody isotypes

The test antigens HIV-1 BG505 T332N SOSIP gp140, HIV-1 CNE55 SOSIP gp140, and H1N1 Influenza A/New Caledonia/30/1999 hemagglutinin were enzymatically labeled using biotin ligase (BirA500, Avidity LLC). SARS-CoV-2 D614G Spike was chemically biotinylated with the EZ-Link Sulfo-NHS-Biotinylation Kit (ThermoFisher Scientific). ADCP assays were conducted as previously described (79). Briefly, biotinylated antigens were covalently coupled to fluorescent neutravidin beads. Test and control antibodies were serially diluted threefold from 50 µg/mL across five dilutions. Immune complexes were formed by incubating bead-bound antigens with diluted antibodies. These complexes were then incubated with either THP-1 cells (human monocyte cell line, ATCC TIB-201) for IgG antibodies or DMSO-differentiated HL60 cells (neutrophil-like cell line expressing IgA Fc receptors, ATCC CCL-240) for IgA antibodies. Phagocytic uptake was quantified by flow cytometry (BD LSRII), measuring cell fluorescence. The phagocytic response was calculated as the product of mean fluorescence intensity (MFI) and the frequency of phagocytosis-positive cells, normalized to an antibody-negative (PBS) control. Phagocytosis scores were derived by calculating the area under the titration curves for each antibody-antigen pair using the trapezoidal rule. All experiments were performed in triplicate.

### ADCP assay against non-native HVTN124 antibody isotypes

The BirA biotin-protein ligase bulk lyophilized reaction kit was used to biotinylate avi-tagged proteins to ensure correct orientation of the antigen and coated on fluorescent neutravidin beads as previously described (79, 80). Antigen-coated beads were incubated for 2 hours at 37°C, 5% CO_2_, with a titration of monoclonal antibodies at 2 µg/mL and titrated fivefold. Beads were washed and further incubated with THP-1 cells overnight, fixed with PFA (Sigma), and interrogated on the Cytoflex (Beckman Coulter). ADCP score was calculated as a proportion of THP-1 cells that internalized beads multiplied by the geometric MFI of the population. Signal from a “no antibody” negative control was subtracted to remove the background signal.

### ADCC assay against non-native HVTN124 antibody isotypes

Antibodies were screened for their capacity to cross-link and activate the FcγRIIIa (CD16) receptor on the surface of Jurkat cells and D614G SARS-CoV-2 spike expressing cells or BG505 or NC/99 trimer coated onto plates as a proxy for ADCC. HEK293T cells were transfected with 5 µg of SARS-CoV-2 ancestral variant spike (D614G) using 1 mg/mL PEI-MAX 40,000 (Polysciences) and incubated for 2 days at 37°C. Alternatively, the BG505 protein was coated at 1 µg/mL on a high-binding ELISA 96-well plate and incubated at 4°C overnight. Plates were then washed with PBS and blocked at room temperature for 1 hour with PBS + 2.5% BSA. Subsequently, protein or 1 × 10^5^ spike-transfected cells per well were incubated with monoclonal antibodies (in RPMI 1640 medium supplemented with 10% FBS 1% Pen/Strep (Gibco, Gaithersburg, MD) for 1 hour at 37°C. To confirm spike expression from the HEK293T cells, binding of CR3022 and 946-A6 was detected by anti-IgG APC staining and measured by flow cytometry. Twenty microliter of supernatant was transferred to a white 96-well plate with 50 µL of reconstituted QUANTI-Luc secreted luciferase and read immediately on a Victor 3 luminometer with 1 s integration time. Additionally, 1× cell stimulation cocktail (Thermo Fisher Scientific, Oslo, Norway) and 2 µg/mL ionomycin in R10 were added as controls to induce the transgene and confirm sufficient expression of the Fc receptor.

### Quantification and statistical analysis

ELISA (standard error of the mean; SEM), ADCP/ADCC, and neutralization error bars shown were calculated using GraphPad Prism version 10.4.0.

## Data Availability

Sequences for the four antibodies identified and characterized in this study have been deposited to GenBank under the accession numbers 92412-37791 (heavy chain: PX056135, light chain: PX056136), 92421-33628 (heavy chain: PX056137, light chain: PX056138), 92422-98105 (heavy chain: PX056139, light chain: PX056140), 92422-11670 (heavy chain: PX056141, light chain: PX056142) and will become publicly available once accession formalization resumes. This paper does not report original code. Any additional information required to reanalyze the data reported in this paper is available from the lead contact upon request.

## AFTER EPUB

*[This article was published on 10 November 2025 with Robert H. Carnahan's name misspelled in the byline. The byline was updated in the current version, posted on 25 November 2025.]*

## SUPPLEMENTAL MATERIAL

Supplemental figures jvi.01256-25-s0001.pdf Figures S1 to S12.

ASM does not own the copyrights to Supplemental Material that may be linked to, or accessed through, an article. The authors have granted ASM a non-exclusive, world-wide license to publish the Supplemental Material files. Please contact the corresponding author directly for reuse.
