# Supplementary material for: Glycan-reactive antibodies isolated from human HIV-1 vaccine trial participants show broad pathogen cross-reactivity
Source: J Virol. 2025 Nov 10;99(12):e01256-25. doi: 10.1128/jvi.01256-25 (PMC12724313; doi:10.1128/jvi.01256-25)
Supplement: Supplemental figures — Figures S1 to S12. [file jvi.01256-25-s0001.pdf]

Figure S1

Heavy Chain

	FWR1	CDR1	FWR2	CDR2	FWR3	CDR3	FWR4
92412-37791	EVQLLESGGGLVQPGGSLTLSCAAS	GFIFSRCT	MNWVRQAPGKGLEWVSS	IDGAGDT.	HYQDSVKGRFAISRDDSKSTVYLQMNNLRADDTALYYC	ANRLEEHFDH....	WGQGALVTVAS
92421-33628	EVQLVESGGGLVQPGGSLRLSCVAS	GFIFSQDD	MKWVRQAPGKGLEWVSQ	ISFSGSSK	YYADSVKGRFSISRDNANNALYLQMNSLGDDDTAVYYC	ARAHRSG.......	WGQGTLVTVSS
92422-98105	QVHLDQSGAEVKKPGASVKISCEAS	GYIFSDYF	MHWVRQAPGQGLEWLGM	INSGSGST	DYEQKFHGRISMATDTATHTIYMELRGLRVEDTAVYFC	ARADMQYFDV....	WGPGTLVTVSS
92422-11670	QVHLVESGGGVVQPGASLKLSCTGS	TFSFNKYA	MNWVRQAPDKGLEWLAL	ISSDGNSR	HYADSVNGRLTISRDDSRHSLFLEIHSLRLEDTGVYFC	APDLDSSPSYFWGY	WGQGTLVNVSS

Light Chain

	FWR1	CDR1	FWR2	CDR2	FWR3	CDR3	FWR4
92412-37791	EIVMTQTPLSSPVTLGQPAAISCRSS	QSLVHSNGNTY	LSWLQQRPGQPPRLLIY	KIF	NRFSGVPDRFSGSGAGTDFTLKISRVEAEDVGVYYC	MQATHFPWS	FGQGTKVEIK
92421-33628	EIVLTQSPGTLSLSPGERATLSCRAS	QSVRSSY....	LAWYQQKPGQAPRLLIY	DTS	YRATGIPDRFSGSGSGTDFSLTISRLEPEDFAVYYC	QQYYSSPFA	FGPGTTVDIK
92422-98105	KIVMTQTPLSSPVTLGQPASISCRSS	KSLVHSNGNTY	LSWLQQRPGQPPRLLIY	EVS	HRFSGVPDRFSGSGAGADFTLTISRVEAEDVGVYYC	MQATDFPRT	FGQGTKVEIR
92422-11670	DYDLTQTPVSSPVTLGQSASISCRSS	QSLVHSNGKTY	LSWLHVRSGQPPRLLIY	QIS	SRFPGVPDRFSGSGASTEFTLKISRVEADDVGIYYC	MQASHFPRT	FGQGTKVEIR

Figure S1. Detailed HVTN124 mAb sequence information.
Amino acid sequences of the framework and CDR regions for the heavy (top) and light (bottom) chains of the four HVTN124 mAbs.

Figure S2

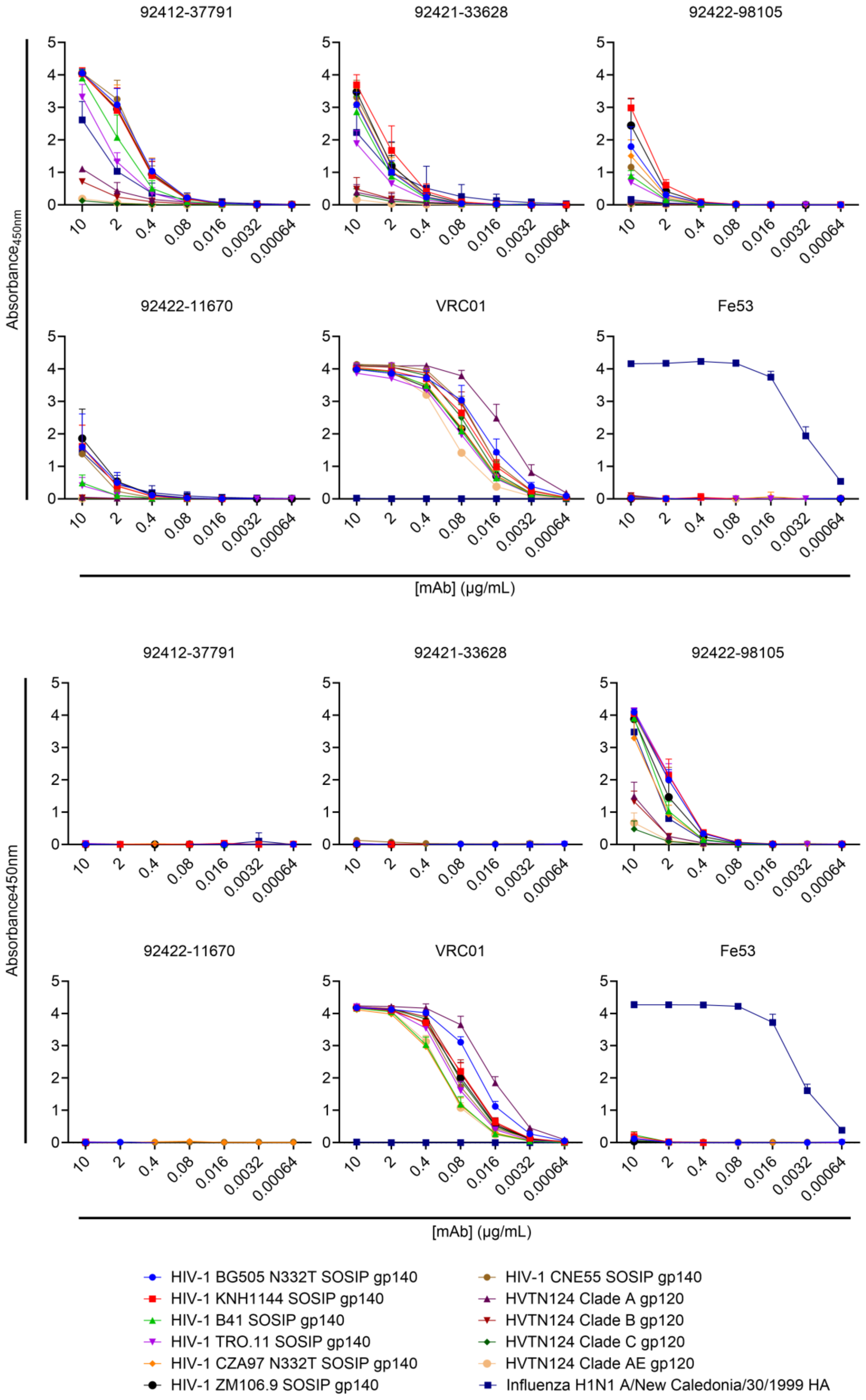

Figure S2. Characterization of HVTN124 mAbs by ELISA.
ELISA validation of HVTN124 mAbs displayed as full curves. ELISA 5-fold curves from a set of three repeats in duplicate are displayed for each of the four HVTN124 mAbs, along with the VRC01 and Fe53 mAb controls, against all 12 antigens used in the LIBRA-seq screening library. Top: Mature HVTN124 mAbs. Bottom: Germline HVTN124 mAbs.

Figure S3

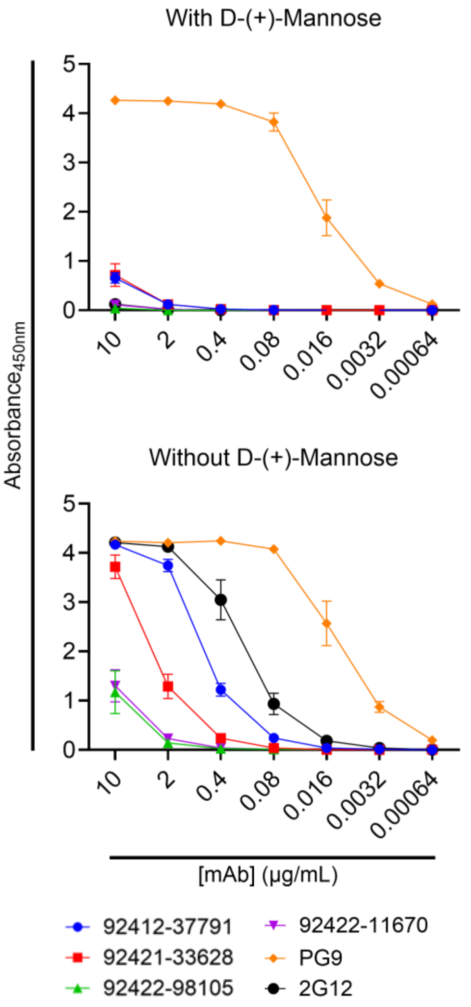

Figure S3. HVTN124 mAbs achieve broad reactivity via N-linked glycan recognition.
Antibody competition with and without 1M D-(+)-Mannose displayed as full curves. Absorbance at 450nm is listed on the Y-axis, while antibody concentration in µg/mL is listed on the X-axis. The four HVTN124 mAbs, along with the V3-glycan-reactive 2G12 and V1/V2-reactive PG9 control mAbs, were incubated with and without 1M D-(+)-Mannose against HIV-1 CNE55 SOSIP gp140. ELISA 5-fold curves for both conditions from a set of three repeats in duplicate are displayed.

Figure S4

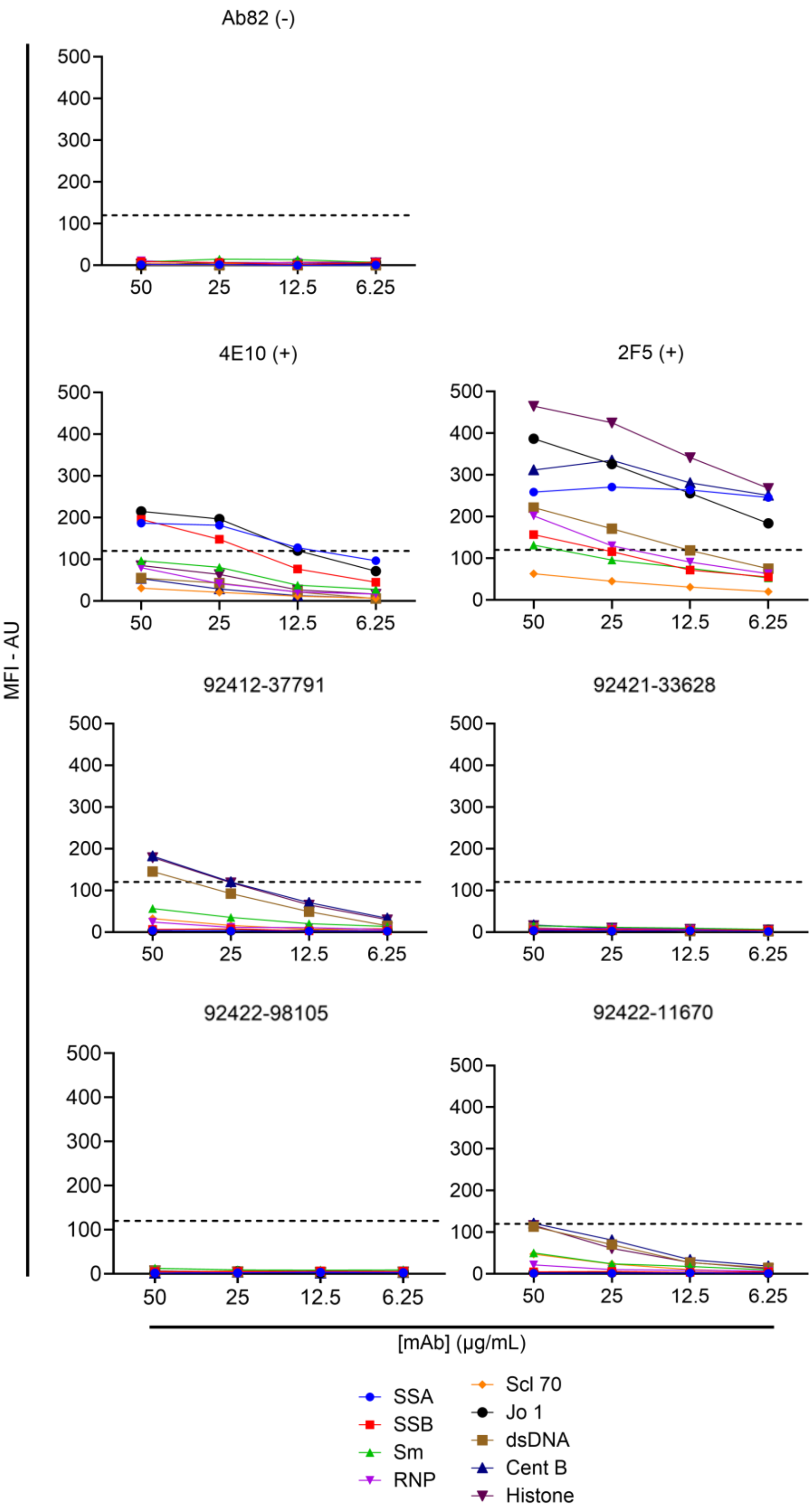

Figure S4. AtheNA autoreactivity analysis of HVTN124 mAbs.
Autoantigen reactivity against the AtheNA panel as full curves. HVTN124 mAbs were tested for autoreactivity against the AtheNA panel. MFI – AU is listed on the Y-axis, while antibody concentration in µg/mL is listed on the X-axis. Positive control antibodies included 4E10 and 2F5, while Ab82 was used as a negative control antibody. Values exceeding 120 MFI at 25 µg/mL for the AtheNA assay are considered positive. Negative MFI – AU values were transformed to zero.

Figure S5

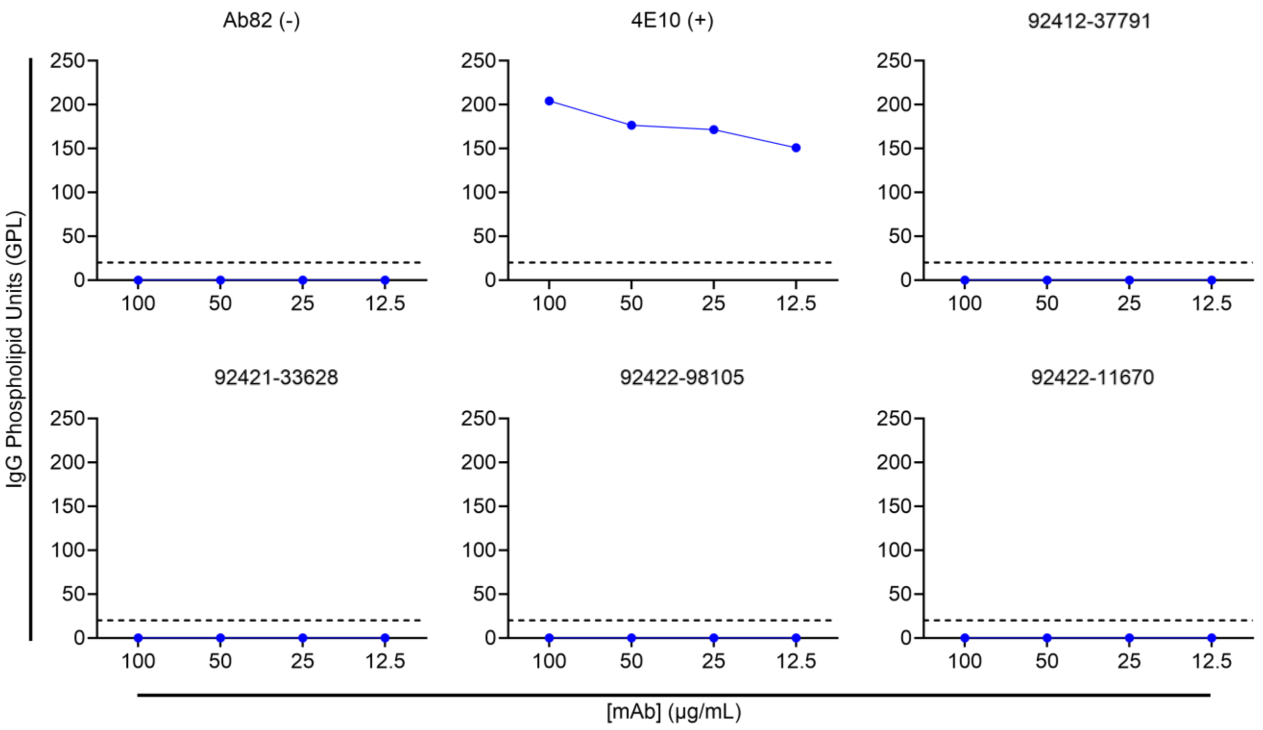

Figure S5. Cardiolipin autoreactivity analysis of HVTN124 mAbs.
Autoreactivity toward cardiolipin as full curves. IgG phospholipid units (GPL) is listed on the Y-axis, while antibody concentration in µg/mL is listed on the X-axis. 4E10 was used as a positive control antibody, while Ab82 was used as a negative control antibody. Values at or greater than 20 GPL at 50 µg/mL are considered positive. Negative GPL values were transformed to zero.

Figure S6

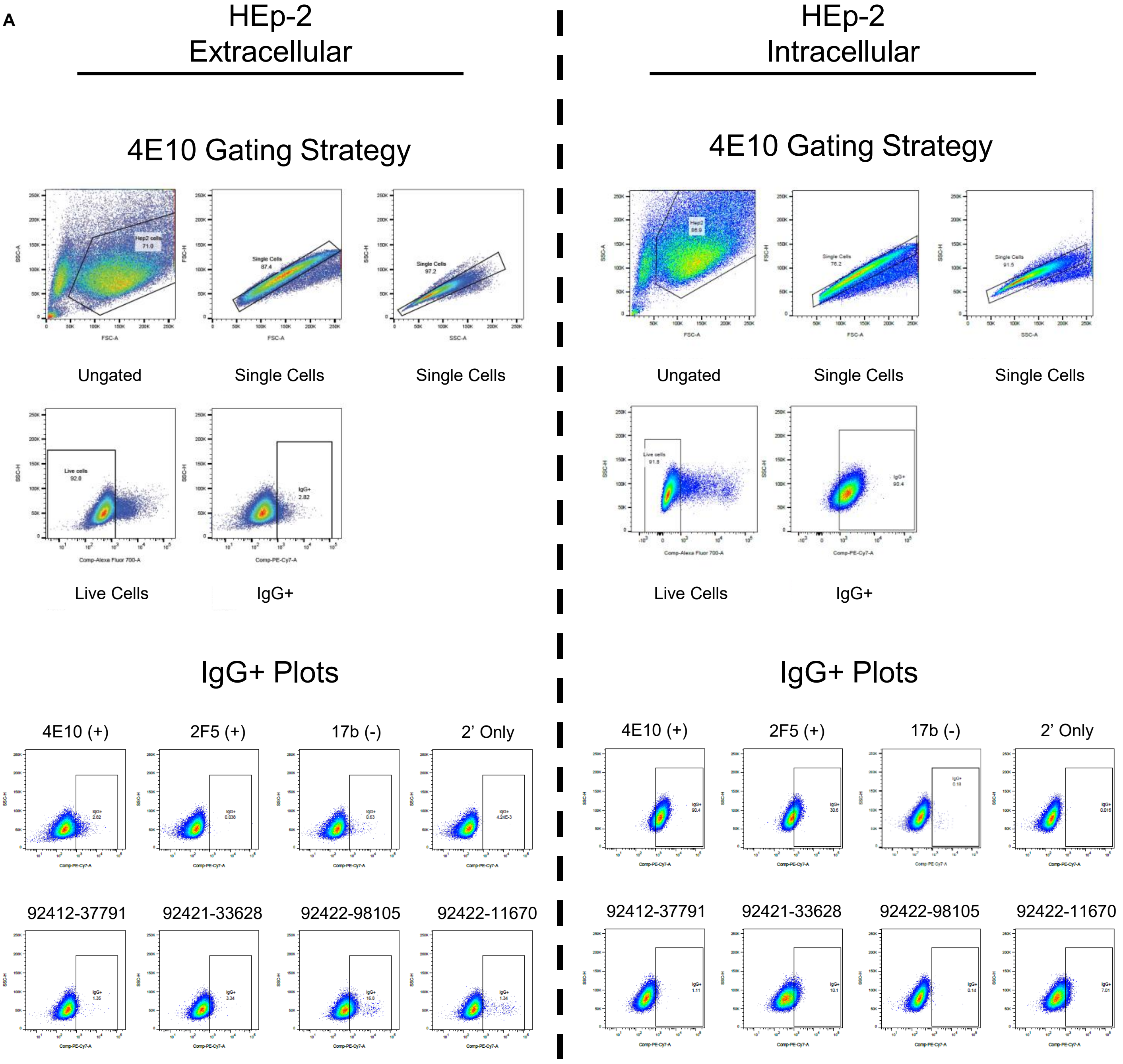

Figure S6. Flow cytometry gating strategies to determine the intracellular and extracellular self-antigen recognition by HVTN124 mAbs.
(A) Left: HEp-2 extracellular gating strategy using 4E10 positive control mAb and IgG+ plots of antibodies. Right: HEp-2 intracellular gating strategy using 4E10 positive control mAb and IgG+ plots of antibodies.

Figure S6

B

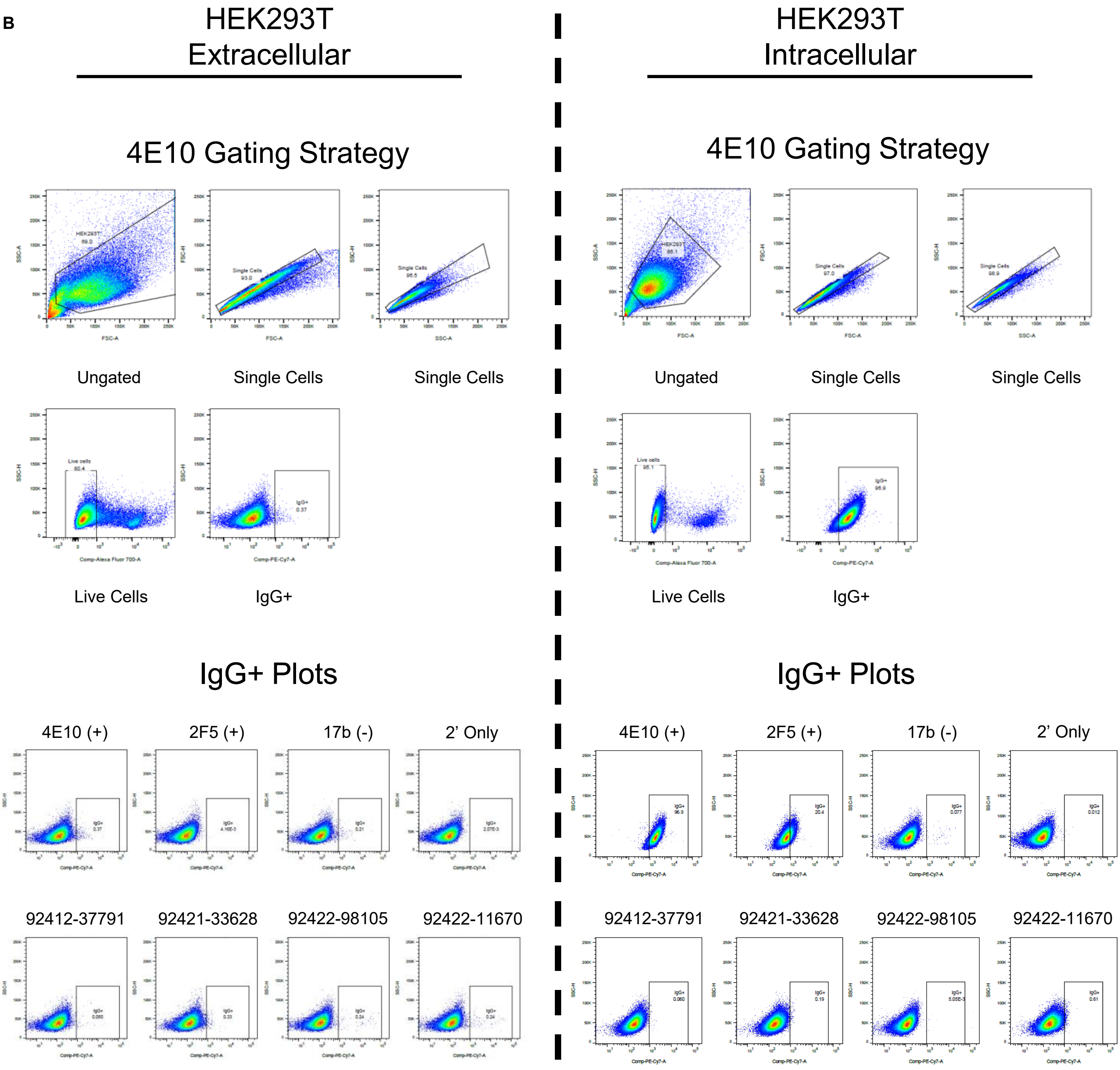

Figure S6. Flow cytometry gating strategies to determine the intracellular and extracellular self-antigen recognition by HVTN124 mAbs.
(B) Left: HEK293T extracellular gating strategy using 4E10 positive control mAb and IgG+ plots of antibodies. Right: HEK293T intracellular gating strategy using 4E10 positive control mAb and IgG+ plots of antibodies.

Figure S6

c

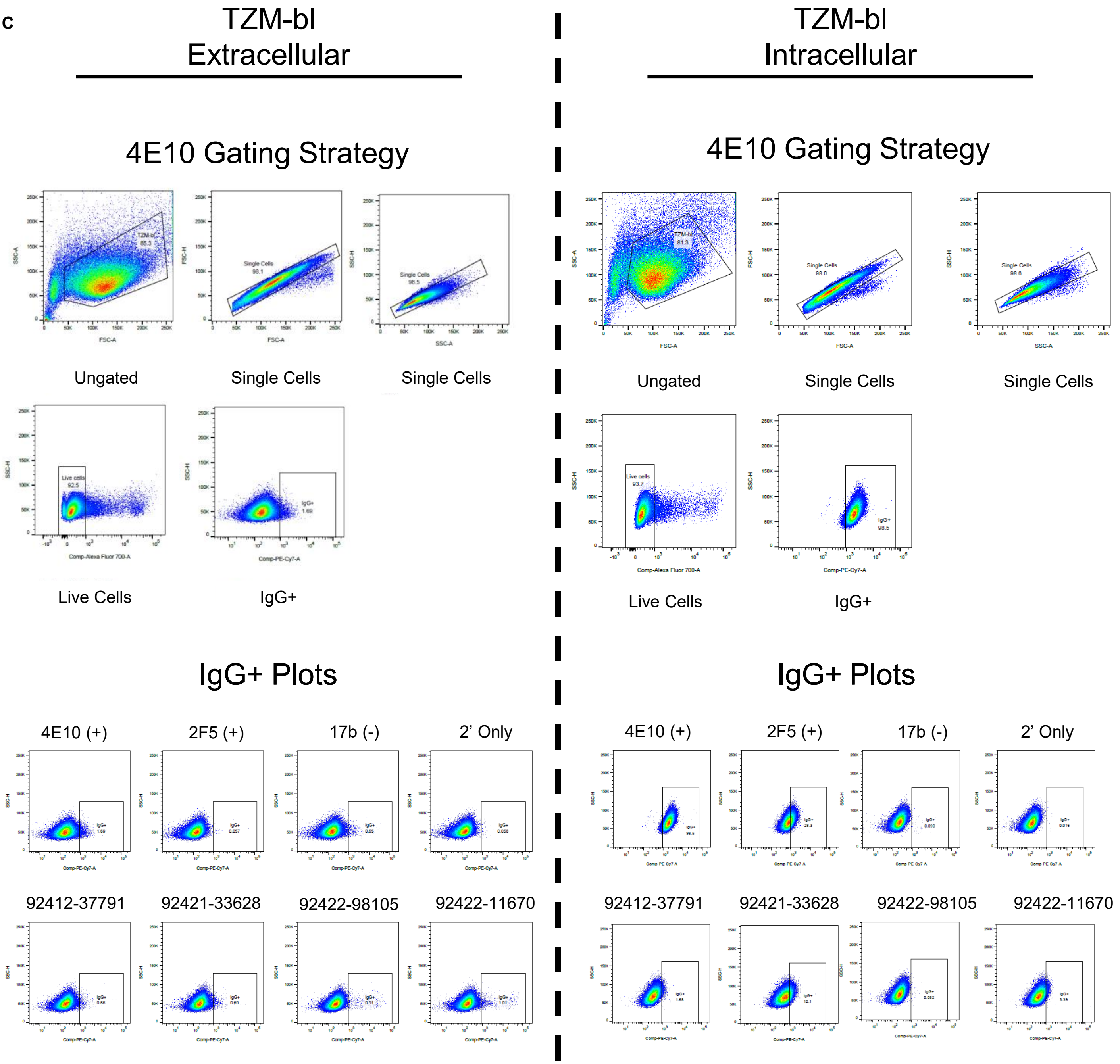

Figure S6. Flow cytometry gating strategies to determine the intracellular and extracellular self-antigen recognition by HVTN124 mAbs.
(C) Left: TZM-bl extracellular gating strategy using 4E10 positive control mAb and IgG+ plots of antibodies. Right: TZM-bl intracellular gating strategy using 4E10 positive control mAb and IgG+ plots of antibodies.

Figure S6

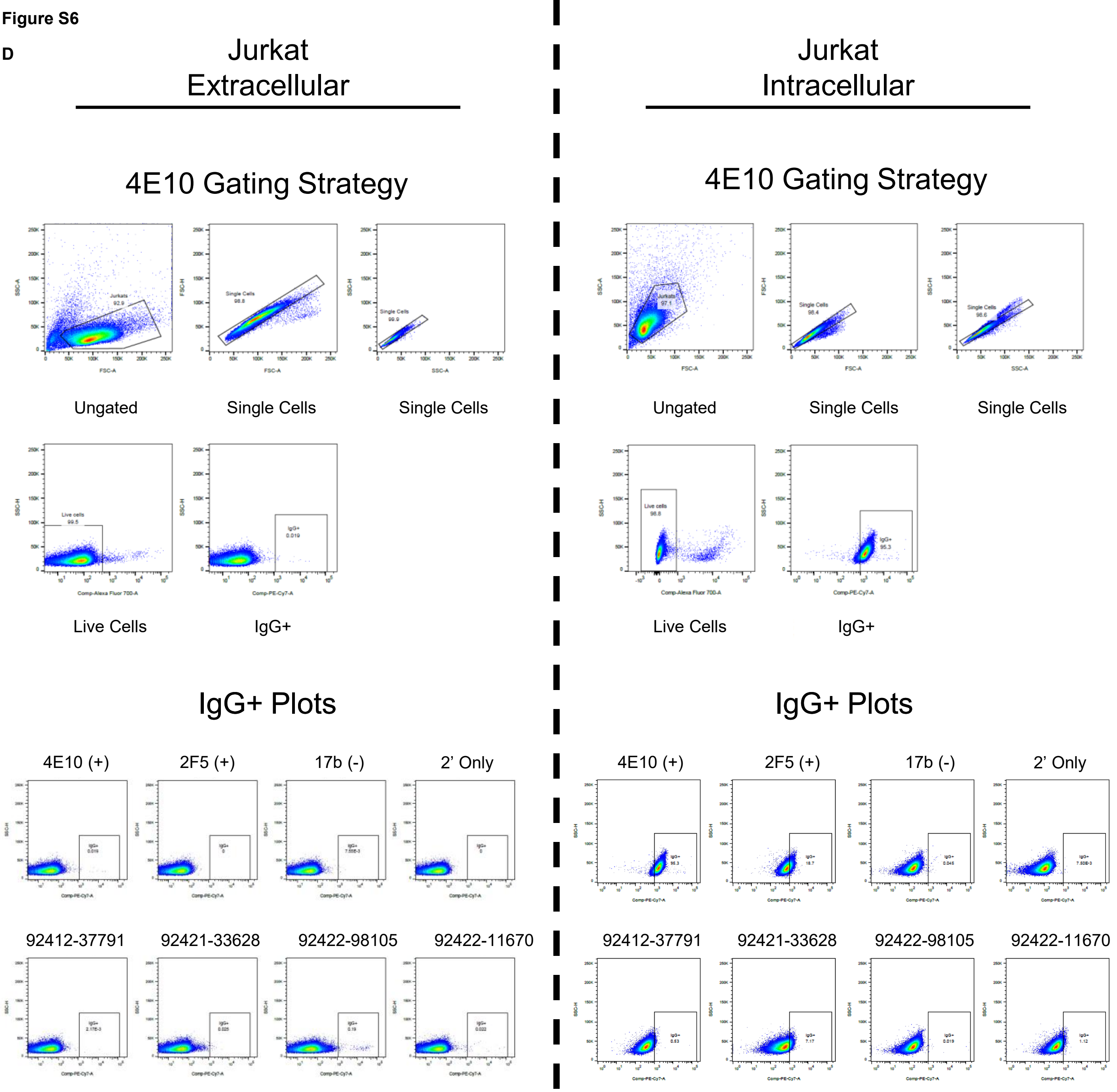

Figure S6. Flow cytometry gating strategies to determine the intracellular and extracellular self-antigen recognition by HVTN124 mAbs.
(D) Left: Jurkat extracellular gating strategy using 4E10 positive control mAb and IgG+ plots of antibodies. Right: Jurkat intracellular gating strategy using 4E10 positive control mAb and IgG+ plots of antibodies.

Figure S6

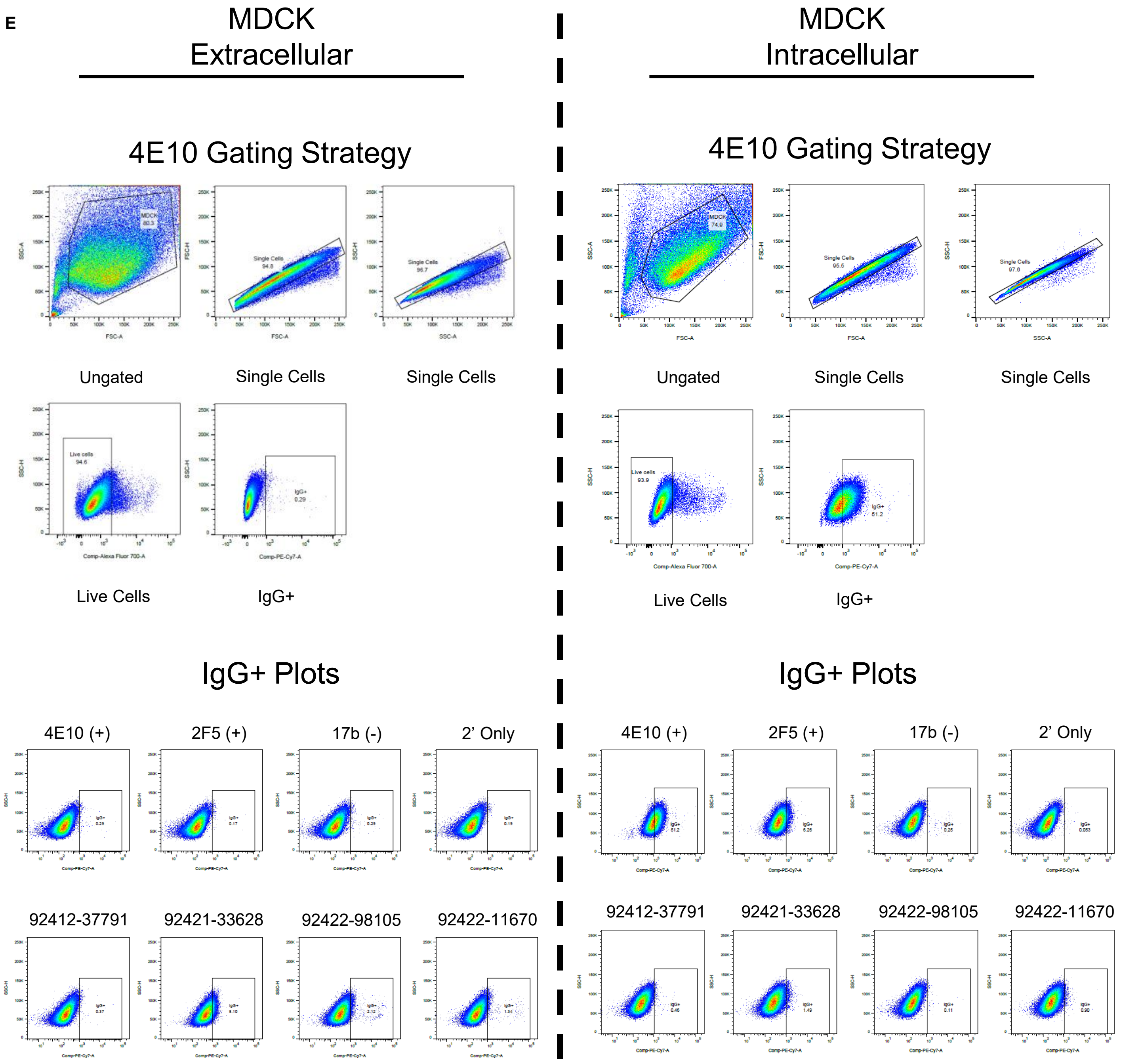

Figure S6. Flow cytometry gating strategies to determine the intracellular and extracellular self-antigen recognition by HVTN124 mAbs.
(E) Left: MDCK extracellular gating strategy using 4E10 positive control mAb and IgG+ plots of antibodies. Right: MDCK intracellular gating strategy using 4E10 positive control mAb and IgG+ plots of antibodies.

Figure S7

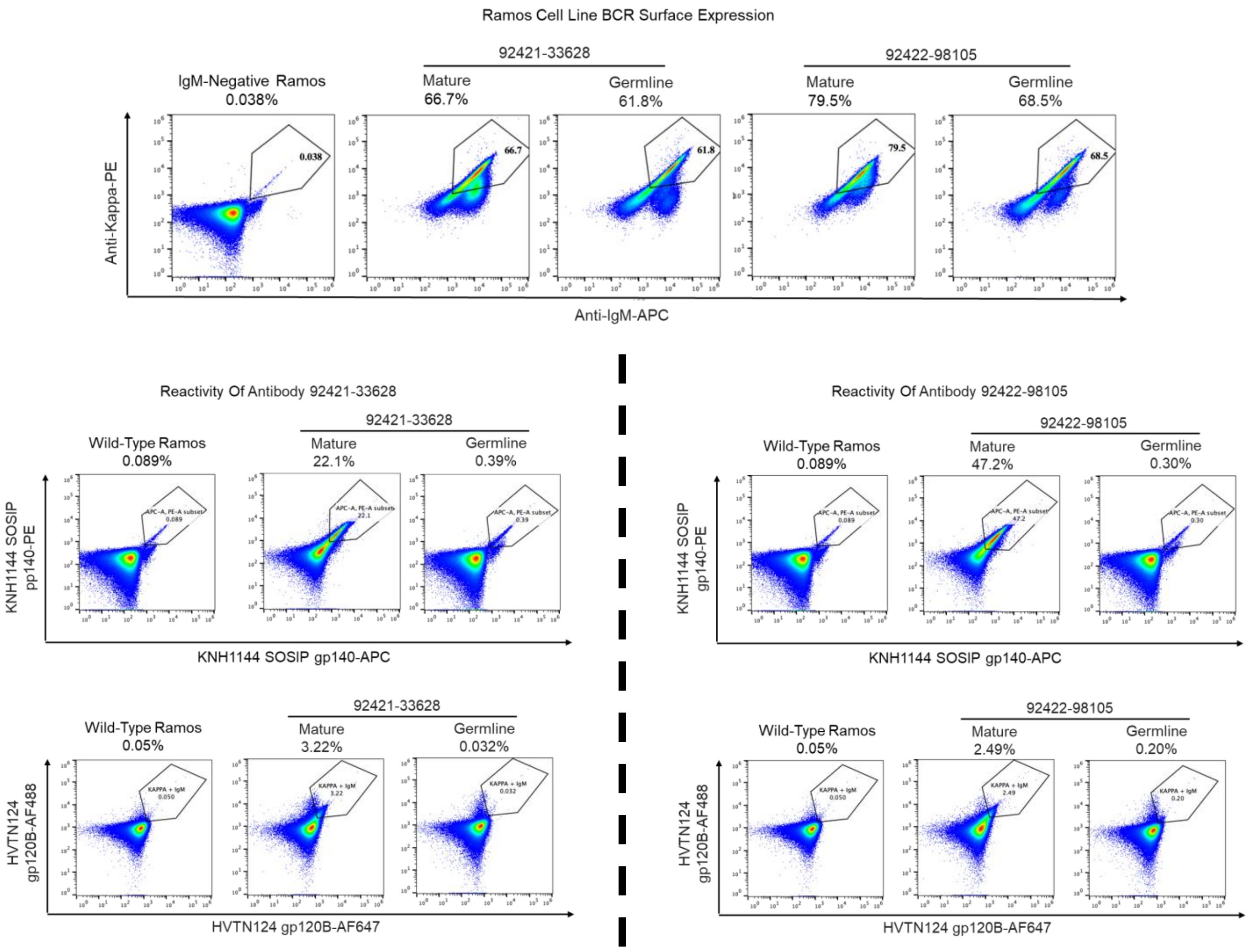

Figure S7. HVTN124 BCR Antigen Interactions
BCR expression of 92421-33628 and 92422-98105 in Ramos cell lines. Ramos cell lines were produced using a previously developed protocol [70]. Top: Confirmation of B cell surface receptors for 92421-33628 and 92422-98105 mature and germline sequences, indicated by IgM+ and Kappa+ populations. Left: Reactivity of mature and germline 92421-33628 against HIV-1 KNH1144 SOSIP gp140 and HVTN124 Clade B gp120. Right: Reactivity of mature and germline 92422-98105 against HIV-1 KNH1144 SOSIP gp140 and HVTN124 Clade B gp120. Percentages of double positive populations are listed below each condition.

Figure S8

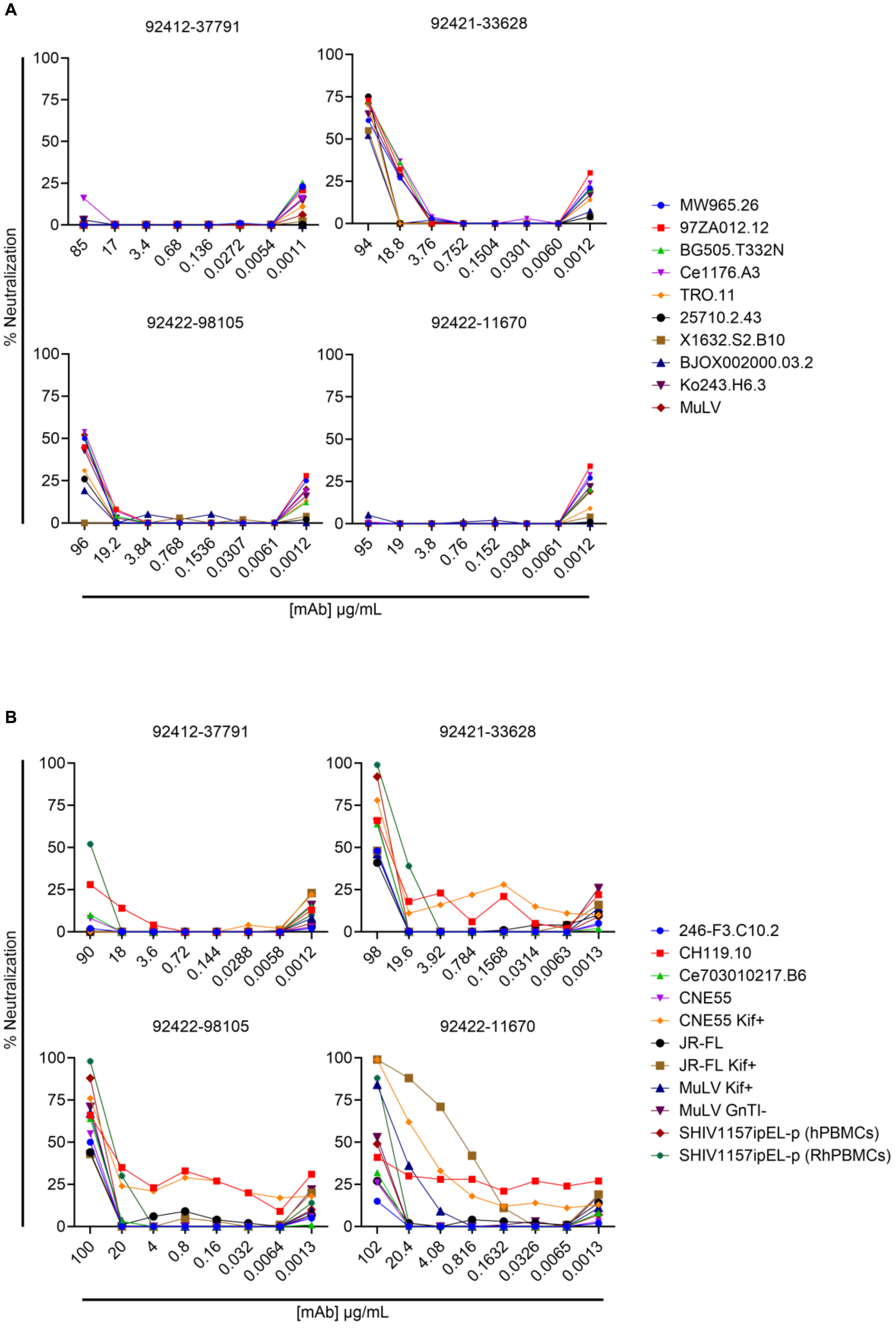

Figure S8. HIV-1 neutralization.

(A) HVTN124 mAbs vs. HIV-1 pseudovirus set 1. Lot 1 of HVTN124 mAbs vs. HIV-1 pseudovirus. Percent neutralization is listed on the Y-axis while antibody concentration in µg/mL is listed on the X-axis. Percent neutralization of the 5-fold curves are displayed for each of the four HVTN124 mAbs being tested against: MMW965.26, 97ZA012.12, BG505.T332N, Ce1176.A3, TRO.11, 25710.2.43, X1632.S2.B10, BJOX002000.03.2, Ko243.H6.3, and MuLV. Negative percent neutralization values were transformed to zero.

(B) HVTN124 mAbs vs. HIV-1 pseudovirus set 2. Percent neutralization is listed on the Y-axis while antibody concentration in µg/mL is listed on the X-axis. Percent neutralization of the 5-fold curves are displayed for each of the four HVTN124 mAbs being tested against: 246-F3.C10.2, CH119.10, Ce703010217.B6, CNE55, CNE55 Kif+, JR-FL, JR-FL Kif+, MuLV Kif+, MuLV GnTI-, SHIV1157ipEL-p (hPBMCs), and SHIV1157ipEL-p (RhPBMCs). Negative percent neutralization values were transformed to zero.

Figure S9

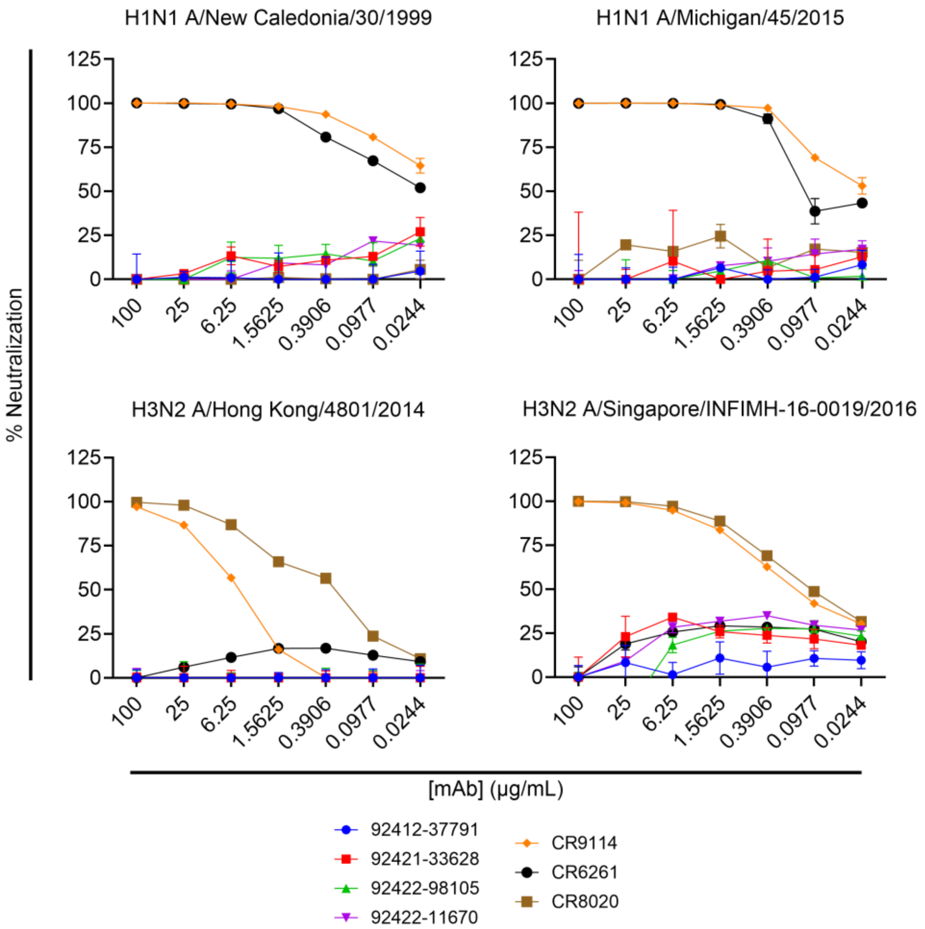

Figure S9. Influenza neutralization.
Neutralization of HVTN124 mAbs vs. H1N1 and H3N2 Influenza viruses as 4-fold curves. HVTN124 mAbs were tested for neutralizing activity against an influenza viral panel that included: H1N1 A/New Caledonia/30/1999, H1N1 A/Michigan/45/2015, H3N2 A/Singapore/INFIMH-16-0019/2016, and H3N2 A/Hong Kong/4801/2014. Of the control mAbs used, CR9114 is known to broadly neutralize influenza A and B viruses, while CR6261 targets group 1 influenza A viruses such as H1N1 isolates, and CR8020 targets group 2 influenza A viruses such as H3N2 isolates. The percent neutralization is listed on the Y-axis, while the antibody concentrations in μg/mL is listed on the X-axis. Negative percent neutralization values were transformed to zero.

Figure S10

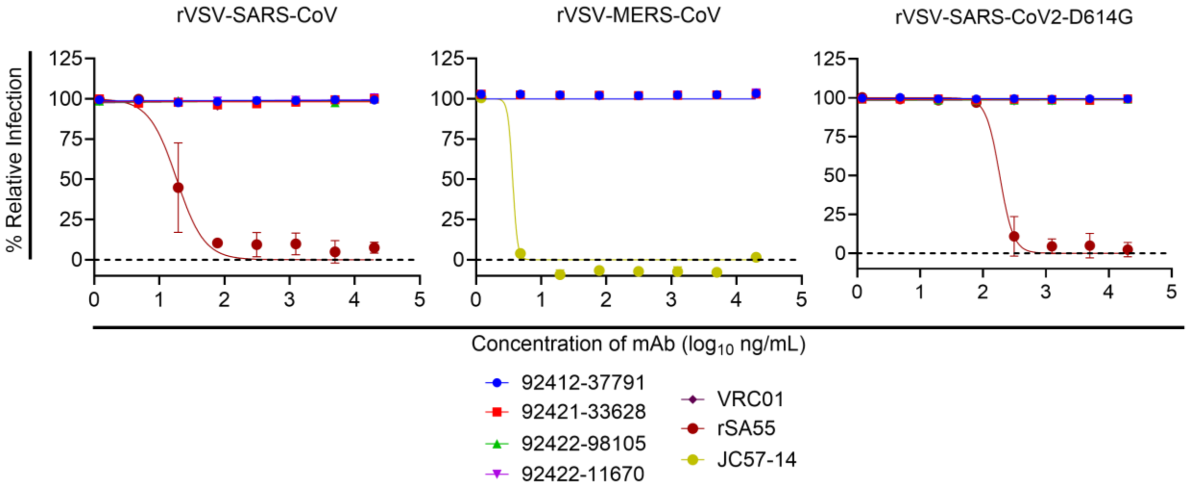

Figure S10. Coronavirus neutralization.
Coronavirus neutralization as 4-fold curves. HVTN124 mAbs were screened for neutralizing activity against rVSV-SARS-CoV-2 D614G, rVSV-MERS-CoV, and rVSV-SARS-CoV. Positive controls included rSA55 for rVSV-SARS-CoV-2 D614G and rVSV-SARS-CoV, while JC57-14 was used for rVSV-MERS-CoV. VRC01 was also included as a negative control mAb. The percent infectivity is listed on the Y-axis, while the antibody concentrations in µg/mL mAb is listed on the X-axis. Data are mean ± standard deviations (SD) of technical triplicates from a representative experiment repeated twice.

Figure S11

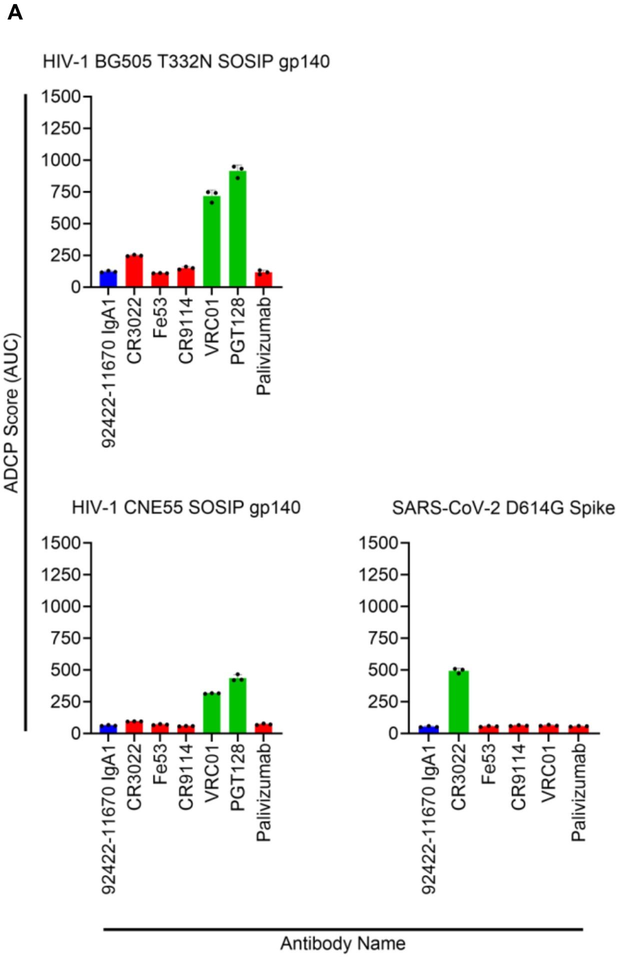

Figure S11. HVTN124 mAb ADCP activity.
(A) HVTN124 ADCP activity using native IgA1 isotype. ADCP activity was measured against HIV-1 BG505 T332N SOSIP gp140, HIV-1 CNE55 SOSIP gp140, and SARS-CoV-2 D614G Spike. Antigens were each biotinylated and conjugated to fluorescent neutravidin beads, then mixed with each mAb. Antibodies are listed along the X-axis, while the Y-axis lists the ADCP score as AUC. The control mAbs used include: VRC01, PGT128 = HIV-1; CR3022 = SARS-CoV-2. Palivizumab was included as a negative control antibody. Antibodies are color coded by HVTN124 mAbs (blue), positive control mAbs (green), and negative control mAbs (red). DMSO-differentiated HL60 cells were subsequently added and ADCP was calculated based on the engulfment of the antigen-coated beads by the THP-1 cells as measured by flow cytometry. Antigen engulfment is indicated as an ADCP score. All experiments were performed in triplicate.

Figure S11

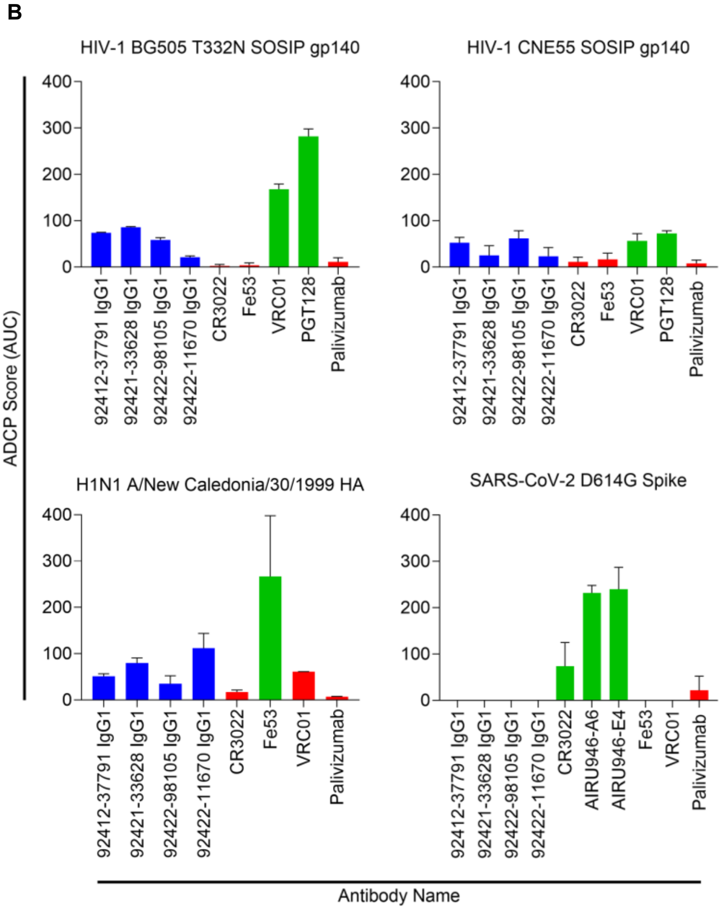

Figure S11. HVTN124 mAb ADCP activity.
(B) HVTN124 ADCP activity using non-native isotype. ADCP activity was measured against HIV-1 BG505 T332N SOSIP gp140, HIV-1 CNE55 SOSIP gp140, influenza H1N1 A/New Caledonia/30/1999 HA, and SARS-CoV-2 D614G Spike. Antigens were each biotinylated and conjugated to fluorescent beads with immobilized streptavidin, then mixed with each mAb. Antibodies are listed along the X-axis, while the Y-axis lists the ADCP score as AUC. The control mAbs used include: VRC01, PGT128 = HIV-1; Fe53 = influenza; CR3022, AIRU946-A6, AIRU946-E4 = SARS-CoV-2. Palivizumab was included as a negative control antibody. Antibodies are color coded by HVTN124 mAbs (blue), positive control mAbs (green), and negative control mAbs (red). THP-1 monocytes were subsequently added and ADCP was calculated based on the engulfment of the antigen-coated beads by the THP-1 cells as measured by flow cytometry. Antigen engulfment is indicated as an ADCP score.

Figure S12

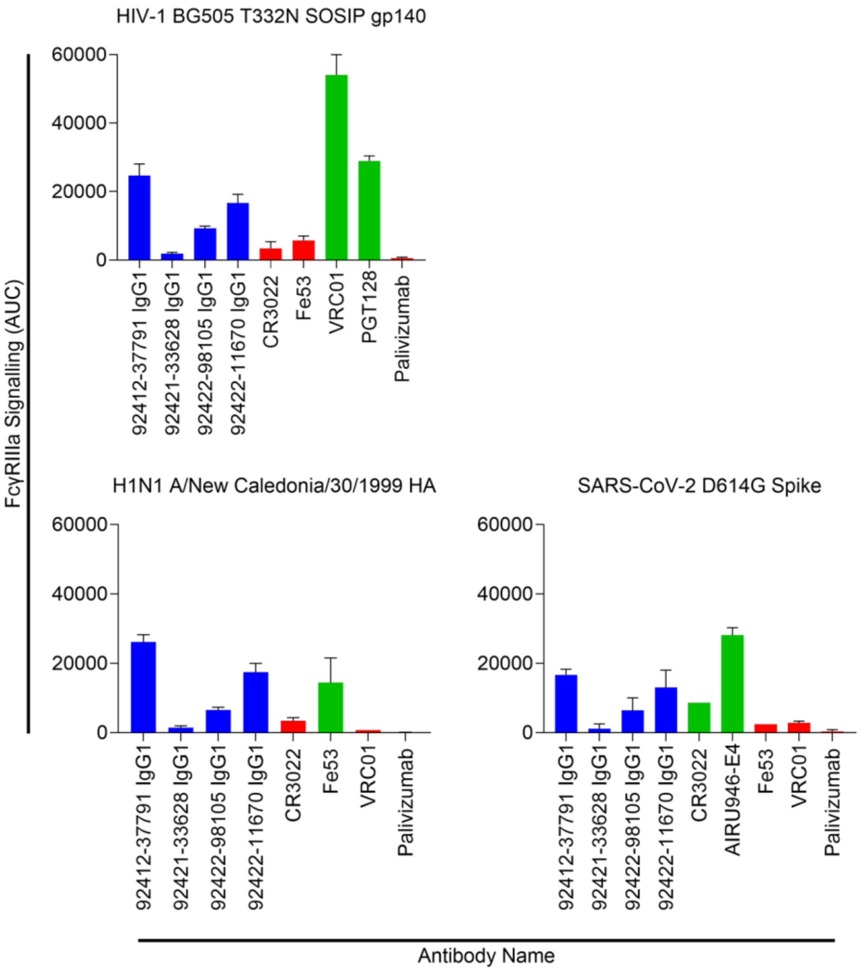

Figure S12. HVTN124 mAb ADCC activity.

HVTN124 ADCC activity using non-native isotype. ADCC activity was measured against HIV-1 BG505 T332N SOSIP gp140, influenza H1N1 A/New Caledonia/30/1999 HA, and SARS-CoV-2 D614G Spike. Antibodies are listed along the X-axis, while the Y-axis lists the FcγRIIIa signaling as AUC. The control mAbs used include: VRC01, PGT128 = HIV-1; Fe53 = influenza; CR3022, AIRU946-E4 = SARS-CoV-2. Palivizumab was included as a negative control antibody. Antibodies are color coded by HVTN124 mAbs (blue), positive control mAbs (green), and negative control mAbs (red). For IgG isotypes. Fc crosslinking activity is reported as AUC. ADCC was calculated based on FcγRIIIa activation on Jurkat cells against HEK293T cells expressing SARS-CoV-2 D614G or against HIV-1 BG505 T332N SOSIP gp140 and H1N1 A/New Caledonia/30/1999 HA trimer coated on to plates.
